# Interrogating and Predicting Tolerated Sequence Diversity in Protein Folds: Application to *E. elaterium* Trypsin Inhibitor-II Cystine-Knot Miniprotein

**DOI:** 10.1371/journal.pcbi.1000499

**Published:** 2009-09-04

**Authors:** Jennifer L. Lahti, Adam P. Silverman, Jennifer R. Cochran

**Affiliations:** Department of Bioengineering, Cancer Center, Bio-X Program, Stanford University, Stanford, California, United States of America; University of Houston, United States of America

## Abstract

Cystine-knot miniproteins (knottins) are promising molecular scaffolds for protein engineering applications. Members of the knottin family have multiple loops capable of displaying conformationally constrained polypeptides for molecular recognition. While previous studies have illustrated the potential of engineering knottins with modified loop sequences, a thorough exploration into the tolerated loop lengths and sequence space of a knottin scaffold has not been performed. In this work, we used the *Ecballium elaterium* trypsin inhibitor II (EETI) as a model member of the knottin family and constructed libraries of EETI loop-substituted variants with diversity in both amino acid sequence and loop length. Using yeast surface display, we isolated properly folded EETI loop-substituted clones and applied sequence analysis tools to assess the tolerated diversity of both amino acid sequence and loop length. In addition, we used covariance analysis to study the relationships between individual positions in the substituted loops, based on the expectation that correlated amino acid substitutions will occur between interacting residue pairs. We then used the results of our sequence and covariance analyses to successfully predict loop sequences that facilitated proper folding of the knottin when substituted into EETI loop 3. The sequence trends we observed in properly folded EETI loop-substituted clones will be useful for guiding future protein engineering efforts with this knottin scaffold. Furthermore, our findings demonstrate that the combination of directed evolution with sequence and covariance analyses can be a powerful tool for rational protein engineering.

## Author Summary

The use of engineered proteins in medicine and biotechnology has surged in recent years. An emerging approach for developing novel proteins is to use a naturally-occurring protein as a molecular framework, or scaffold, wherein amino acid mutations are introduced to elicit new properties, such as the ability to recognize a specific target molecule. Successful protein engineering with this strategy requires a dependable and customizable scaffold that tolerates modifications without compromising structure. An important consideration for scaffold utility is whether existing loops can be replaced with loops of different lengths and amino acid sequences without disrupting the protein framework. This paper offers a rigorous study of the effects of modifying the exposed loops of *Ecballium elaterium* trypsin inhibitor II (EETI), a member of a family of promising scaffold proteins called knottins. Through our work, we identified sequence patterns of modified EETI loops that are structurally tolerated. Using bioinformatics tools, we established molecular guidelines for designing peptides for substitution into EETI and successfully predicted loop-substituted EETI variants that retain the correct protein fold. This study provides a basis for understanding the versatility of the knottin scaffold as a protein engineering platform and can be applied for predictive interrogation of other scaffold proteins.

## Introduction

Protein-protein interactions govern many biological processes in the cell, often with high affinity and specificity. Such interactions are typically mediated by a relatively small portion of the protein, while the remainder of the molecule serves as a framework to ensure the proper presentation of the binding epitopes. Many naturally-occurring proteins with diverse functions are based on common protein frameworks; for example, the immunoglobulin fold is a widespread structural motif found in antibodies, enzymes, and receptors. These common protein frameworks, or molecular scaffolds, can be engineered for novel properties, such as altered molecular recognition [1], increased stability [2], or improved expression levels [3], through the incorporation or evolution of functional epitopes. Ideally, molecular scaffolds should have high intrinsic conformational stabilities and be structurally tolerant of sequence modifications, including insertions, deletions, or substitutions. While antibodies are the most developed class of molecular scaffold, their application is limited in many cases by their large size, complex fold, cost-intensive manufacturing, and complicated patent considerations [4],[5]. Thus, in the past decade there has been much effort toward developing non-antibody scaffolds with enhanced structural robustness, ease of modification, and cost-efficient production. Examples of such alternative molecular scaffolds include: fibronectin, protein A, ankyrin repeat proteins, lipocalins, thioredoxin, ribose-binding proteins, protease inhibitors, PDZ domains, and knottins (reviewed in [4]–[7]). These alternative molecular scaffolds have been engineered for applications in biochemical assays [8], separation technologies [9], and diagnostics and therapeutics [4],[10].

Directed evolution of a protein scaffold for new molecular recognition properties is often achieved by screening focused libraries and isolating clones that bind to a target with high affinity. Prior to screening, a library of protein variants is created by replacing one or more existing loops or domains with new sequences in which the amino acids are randomized at a few or all positions. In some examples, such as the thioredoxin aptamer, a single loop has been substituted [11], while in other cases, such as the 10^th^ domain of fibronectin, as many as three loops have been engineered [12]. One major limitation of this approach is that substitution of entire loops or functional domains may lead to misfolding or loss of structural integrity [13]. In addition, while some new loop sequences represented in the library will lead to properly folded and functional proteins, other loop sequences may not be tolerated and will lead to misfolded, aggregated, or otherwise inactive proteins. Moreover, specific residues may be preferred in certain positions while forbidden in others, or the presence of a specific residue in one position may dictate the presence of another specific residue at a nearby position. In addition to positional amino acid preferences, the length of the substituted loop sequence may also be critical for the structural integrity of the protein [14]. For example, steric or torsional constraints may prohibit substituting a loop with a peptide of shorter length, while substitution with a longer peptide may be highly destabilizing due to entropic factors.

**Figure 1:**
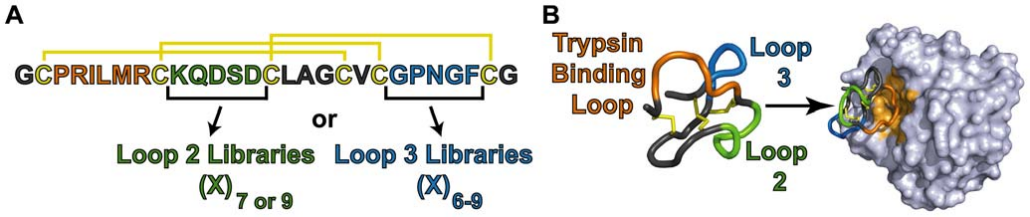
Schematic for interrogating the tolerance of sequence diversity in knottin loops. (A) Six libraries of loop-substituted knottin variants were designed based on the wild-type sequence of EETI. Libraries were created by replacing cysteine-flanked loop 2 (green) or loop 3 (blue) sequences with peptides of randomized amino acids (X) and varying lengths (n). The trypsin binding loop (orange) was not replaced, but instead used as a handle to evaluate the proper folding of EETI loop-substituted clones. Disulfide bonds are shown in yellow. (B) The binding interaction between trypsin (light grey) and EETI (PDB 2eti and 1h9h) is mediated through the trypsin binding loop, and is dependent on the correct formation of all three disulfide bonds. This interaction was exploited for high-throughput isolation of properly folded EETI loop-substituted variants.

A better understanding of the tolerated loop lengths and compositional parameters of a protein would be helpful for evaluating its utility as a scaffold; such insight would allow for the creation of optimal focused libraries and the prediction of admissible sequence modifications that lead to correct protein folding. Here, we describe a comprehensive study on the tolerance of scaffold loop substitution with different sequences and loop lengths using a small, highly structured polypeptide, the *Ecballium elaterium* trypsin inhibitor II (EETI, UniprotKB/Swiss-Prot P12071, Figure 1A). Further, our work applies the findings from the study of EETI loop tolerance to the prediction of artificial, loop-substituted knottin sequences that yield properly folded proteins. This novel approach toward interrogating functional tolerance in a predictive manner is useful not only for the EETI scaffold, but also for the creation of optimally-designed libraries of scaffold proteins in general.

EETI belongs to the cystine-knot (knottin) family of proteins [15], a class of small polypeptides (typically 20–60 amino acids) that possess several advantageous characteristics for their development as molecular scaffolds [7]. Knottins contain three disulfide bonds interwoven into a molecular ‘knot’ that constrain loop regions to a core of anti-parallel β-sheets. The unique topology of the knottin fold imparts high chemical and thermal stability [16] and resistance to proteolysis [17], which are important for biotechnology and biomedical applications. Moreover, knottins can be chemically synthesized and folded *in vitro* [18] or produced recombinantly in various expression systems [19]–[22]. As a prototypical member of the knottin family, the folding pathway and structure of EETI have been well studied [23]–[25]. EETI is composed of 28 amino acids with three disulfide-constrained loops: loop 1 (the trypsin binding loop, residues 3–8), loop 2 (residues 10–14), and loop 3 (residues 22–26) (Figure 1A). Although the inhibition of trypsin is mediated exclusively through binding of EETI loop 1 (Figure 1B), disruption of any of the three disulfide bonds will abolish the EETI-trypsin interaction [26]. EETI has been the subject of previous mutagenesis studies aimed at investigating its protein fold [26] or altering its binding specificity through introducing diversity into loop 1[27]–[29]. While these previous studies support the potential for using EETI as a molecular scaffold, substitution of loops 2 and 3 has not been well explored, and the tolerated sequence space for these loops is unknown.

Here we present a novel combinatorial and computational approach for interrogating functional tolerance of a molecular scaffold in a predictive manner. We created libraries of EETI mutants where loop 2 or loop 3 was substituted with randomized sequences of varying lengths, and used high-throughput screening to identify clones that were properly folded based on their ability to bind to trypsin (Figure 1). We then performed a detailed bioinformatics analysis on the sequences of isolated trypsin-binding mutants, and used this information to successfully predict new loop sequences that led to properly folded EETI variants.

## Results

### Display of EETI loop-substituted libraries on the surface of yeast

**Figure 2:**
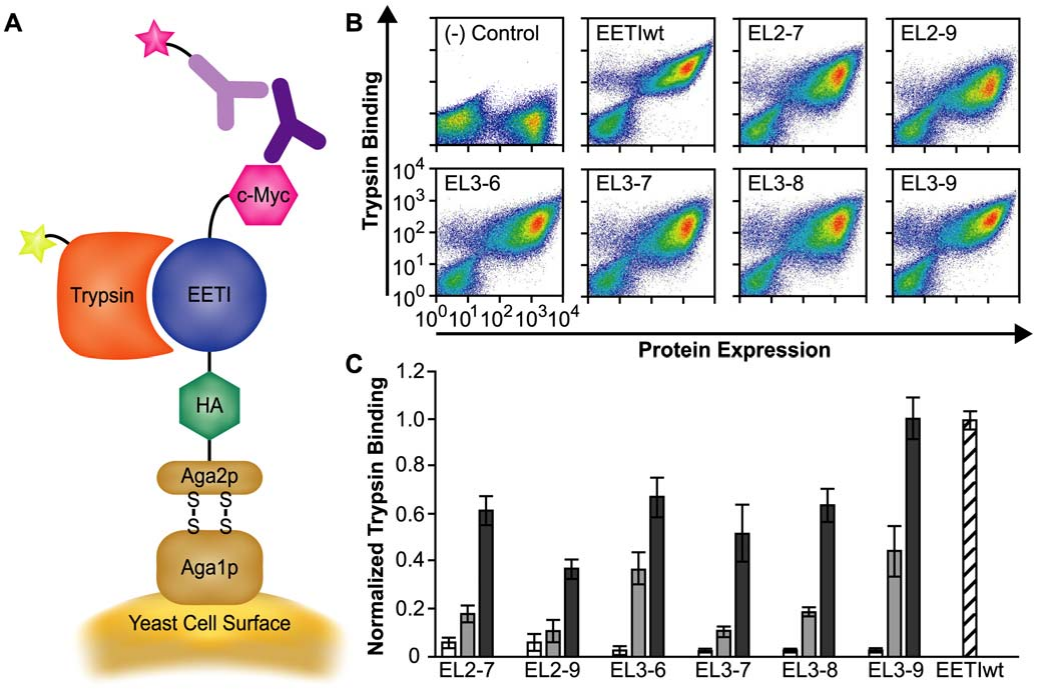
Yeast surface display of EETI knottins and isolation of properly folded EETI loop-substituted clones. (A) Wild-type EETI or loop-substituted clones were displayed on the surface of yeast as fusion proteins to the Aga2p yeast mating subunit and were flanked by N-terminal HA and C-terminal cMyc epitope tags. Protein expression on the yeast cell surface was detected by flow cytometry with antibodies against the cMyc epitope tag, and proper folding of the displayed proteins was determined by binding to fluorescently-labeled trypsin. (B) Yeast-displayed libraries of EETI loop-substituted proteins were enriched for properly folded clones using dual-color fluorescence-activated cell sorting. Density plots depict protein expression (x-axis) versus trypsin binding (y-axis) for enriched pools of loop-substituted clones after four rounds of sorting. EETIwt and potato carboxypeptidase inhibitor, a knottin that does not bind trypsin, are shown as positive and negative controls, respectively. (C) The progression of enrichment for properly folded EETI loop-substituted variants was monitored by flow cytometry and quantified by trypsin binding levels (at 25 nM), normalized for differences in protein expression levels. Unsorted libraries (white), libraries after two rounds of sorting (light grey), and libraries after four rounds of sorting (dark grey), as compared to EETIwt (striped). Trypsin-binding experiments were performed in triplicate and error bars denote standard deviations.

Wild-type EETI (EETIwt) was displayed on the yeast cell surface as an N-terminal protein fusion to the yeast Aga2p agglutinin subunit [30]. A schematic of the yeast display platform is shown in Figure 2A. We measured cell surface expression levels of the EETIwt fusion protein by flow cytometry, after staining yeast cells with a primary antibody against a C-terminal cMyc epitope tag followed by the addition of a fluorescently-labeled secondary antibody. Next, we used fluorescently-labeled trypsin, the native binding partner of EETIwt, to assay whether yeast-displayed EETIwt was properly folded and functional [26] (Figure 2). We showed that EETIwt was well-expressed on the yeast surface and that fluorescently-labeled trypsin bound specifically to yeast-displayed EETIwt with an approximate equilibrium binding constant of 25 nM (data not shown).

Next, in order to explore the tolerance of the EETI scaffold for different loop sizes and amino acid compositions, we created yeast-displayed loop-substituted libraries in which a single cysteine-flanked loop of EETI was substituted with randomized amino acid sequences of varying lengths (Figure 1A). Libraries were generated by overlap extension PCR using oligonucleotides with degenerate NNS codons (where N = A, T, G, C and S = G or C), which encode for all 20 amino acids and only the TAG stop codon. We generated six libraries in total: two libraries of EETI loop 2 variants with substitution lengths of 7 amino acids (EL2-7) and 9 amino acids (EL2-9), and four libraries of EETI loop 3 variants with substitution lengths of 6 amino acids (EL3-6), 7 amino acids (EL3-7), 8 amino acids (EL3-8), and 9 amino acids (EL3-9). We did not mutate EETI loop 1, which is responsible for binding to trypsin, but instead used it as a handle to probe the structural integrity of the EETI loop-substituted clones (Figure 1B). To create the libraries, mutant DNA was electroporated into the *Saccharomyces cerevisiae* EBY100 strain along with linearized yeast-display plasmid as previously described [31]. By performing dilution plating, we estimated that the sizes of the loop-substituted libraries ranged from 5×10^6^–1×10^7^ transformants each.

### Isolation of EETI loop-substituted trypsin-binding clones

Previous studies showed that trypsin binding can be used as a convenient handle to examine formation of the correct pairings of disulfide-bonded cystine residues in EETI [26]. Therefore, we screened each of the EETI loop-substituted libraries for clones that were both displayed on the yeast cell surface (as detected by antibodies against the C-terminal cMyc epitope tag) and properly folded (as determined by their ability to bind fluorescently-labeled trypsin) using dual-color fluorescence-activated cell sorting (FACS) (Figure 2). We performed multiple rounds of FACS on each yeast-displayed library, each time collecting the 1–2% of clones that were the best displayed and exhibited the highest levels of trypsin-binding. The sorts were performed with this stringency in order to enrich each library to near wild-type EETI trypsin binding levels while maintaining as large a diversity of sequences as possible. After four rounds of sorting, a pool of clones showing moderate to wild-type levels of trypsin binding had been isolated from each library (Figure 2B and C).

### Sequence analysis of EETI loop-substituted libraries

**Figure 3:**
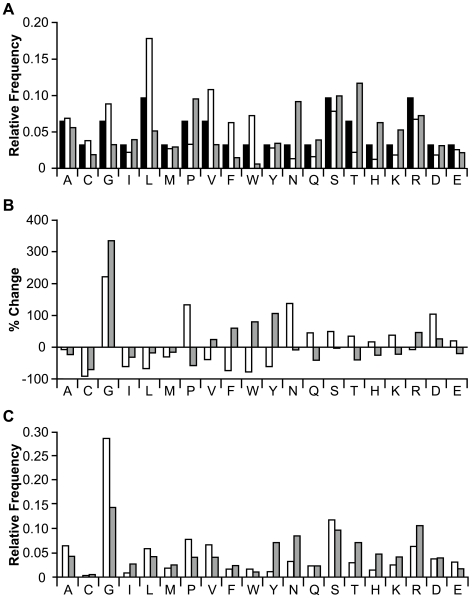
Sequence analysis of randomized peptides from EETI loop-substituted libraries. (A) Amino acid frequencies anticipated from an NNS degenerate codon loop library (black) are shown compared to the observed frequencies of randomized peptides from unsorted EETI loop 2-substituted (white) and EETI loop 3-substituted (grey) libraries. (B) The percent change of individual amino acid frequencies in the substituted loops of enriched EETI loop 2-substituted (white) and EETI loop 3-substituted (grey) clones compared to their frequencies in the respective unsorted libraries. (C) Amino acid frequencies in the randomized peptides of properly folded EETI loop 2-substituted (white) and EETI loop 3-substituted (grey) clones enriched for trypsin binding.

We sequenced at least 50 clones from each of the six original libraries to confirm that the substituted loops were of the correct lengths and had diverse amino acid compositions (Dataset S1). The amino acid frequencies of the loop-substituted regions were generally similar to those expected from a degenerate NNS codon library (Figure 3A). To obtain an analytical measurement of the diversity within each of the loop-substituted libraries, we applied the method of Makowski & Soares [32] to the sequences of clones from each original library. Using the population diversity (POPDIV) algorithm, we determined that the functional diversities (i.e. the percentage of possible library members of a population that are present, adjusted for differences in copy numbers of the members) of the EETI loop-substituted libraries ranged from 3%–11%, corresponding to effective library sizes of 10^6^–10^10^. These functional diversity values are roughly as expected for randomly generated libraries, due to differences in amino acid frequencies resulting from inherent biases in the genetic code [32].

Next, to obtain information on the amino acid composition of properly folded EETI loop-substituted proteins, we sequenced at least 50 clones from each of the libraries after enrichment for trypsin binders (Dataset S1). We examined the sequences from the enriched libraries to determine whether their functional diversities had changed over the course of enrichment for properly folded clones. Using the POPDIV algorithm [32], we found that the functional diversities of the enriched libraries had decreased 10–300 fold. Not surprisingly, libraries that showed the greatest trypsin binding levels after FACS (Figure 2C) also had the greatest decreases in diversity. The amino acid compositions of the enriched library populations also differed from their original, unsorted counterparts (Figure 3B and C). Notably, the frequency of glycine increased in all enriched EETI loop-substituted libraries compared to the starting libraries and cysteine virtually disappeared from all trypsin-binding clones, except in the EL3-7 library (see below) (Figure 3B and C).

### Positional sequence analysis of properly folded EETI loop 2-substituted clones

**Figure 4:**
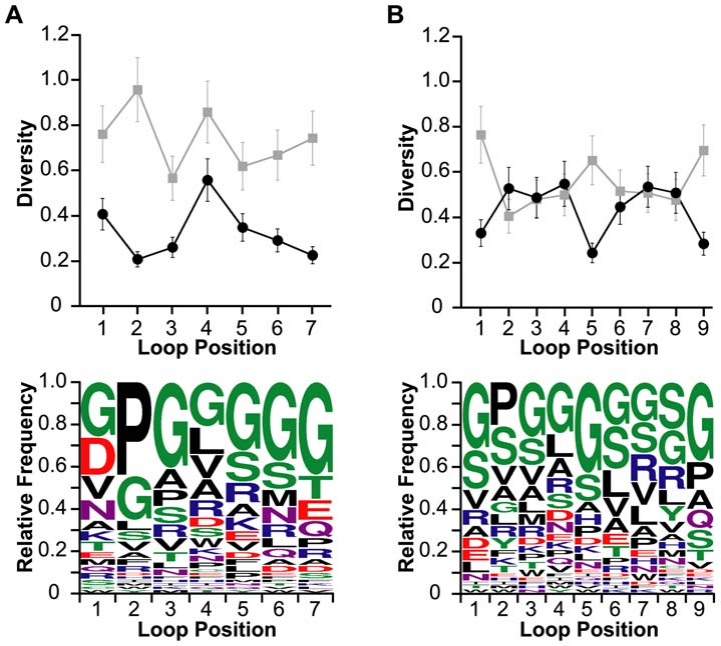
Positional diversities and amino acid preferences of EETI loop 2-substituted clones. Positional diversities of the (A) EL2-7 and (B) EL2-9 libraries before sorting (grey) and after enriching for trypsin-binding clones (black) are depicted in the upper panels. A diversity score of 0.05 denotes complete conservation while a score of 1.0 signifies the presence of all 20 amino acids in equal proportions. Preferred amino acids at each loop-substituted position of enriched libraries are shown in the lower panels, with amino acids colored according to chemical property: polar (green), basic (blue), acidic (red), external polar (purple), and hydrophobic (black).

To quantitatively assess the positional diversities along the lengths of the loop-substituted peptides, we applied the amino acid sequence diversity (DIVAA) algorithm [33] to the sequences of enriched trypsin-binding clones. We found that EETI loop 2-substituted clones isolated from both EL2-7 and EL2-9 libraries were moderately tolerant of substitution across all loop positions, with average diversity scores of 0.3±0.1 and 0.4±0.1 for loop lengths of seven and nine amino acids, respectively (Figure 4). To put this into context, a score of 0.05 indicates complete conservation of a single amino acid and a score of 1.0 indicates the presence of all amino acids in equal proportions. Glycine comprised approximately 25–30% of all amino acids in EETI loop 2-substituted trypsin-binding clones at all but the second loop position. On average, EETI loop 2-substituted sequences of both 7- and 9-amino acids contained approximately 2 glycine residues per clone. Proline residues, which commonly populate turn segments, predominated in the second position of EETI loop 2-substituted variants (Figure 4).

### Positional sequence analysis of properly folded EETI loop 3-substituted clones

**Figure 5:**
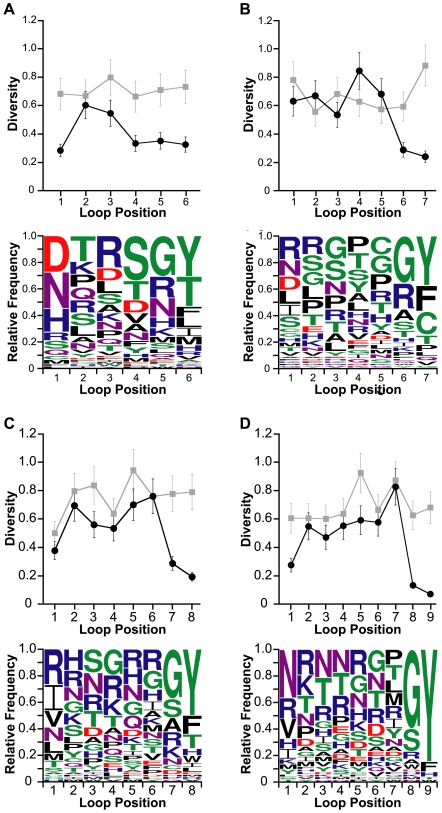
Positional diversities and amino acid preferences of EETI loop 3-substituted clones. Positional diversities of the (A) EL3-6, (B) EL3-7, (C) EL3-8, and (D) EL3-9 libraries before sorting (grey) and after enriching for trypsin-binding clones (black) are depicted in the upper panels. A diversity score of 0.05 denotes complete conservation while a score of 1.0 signifies the presence of all 20 amino acids in equal proportions. Preferred amino acids at each loop-substituted position of enriched libraries are shown in the lower panels, with amino acids colored according to chemical property: polar (green), basic (blue), acidic (red), external polar (purple), and hydrophobic (black).

The overall diversities of EETI loop 3-substituted clones were slightly higher than those of loop 2-substituted clones, with average diversity scores ranging from 0.4±0.1 (for EL3-6 clones) to 0.5±0.2 (for EL3-7 and EL3-8 clones) (Figure 5). The enriched EL3-9 library had an intermediate average diversity score of 0.4±0.2, owing to the high conservation of the final two loop positions (Figure 5). Although the average diversity scores of enriched loop 2- and loop 3-substituted libraries were similar, the amino acid variability for specific positions (positional diversity) in loop 3-substituted clones had a larger range than that observed in loop 2-substituted clones. We found that the greatest levels of diversity occurred in the middle positions of the substituted sequences of loop 3 variants while the first, penultimate, and final positions had the lowest diversities (Figure 5). Nearly half of all trypsin-binding EETI loop 3-substituted clones contained sequences that began with one of three preferred residues: asparagine, arginine, or aspartate (Figure 5). The most common amino acids for the penultimate and final positions of EETI loop 3 were glycine and tyrosine, respectively; nearly a quarter of all loop 3 substituted sequences from enriched clones ended in a glycine-tyrosine doublet. We observed the aforementioned trends in trypsin-binding EETI loop 3-substituted clones across all loop lengths.

EETI loop 3 was tolerant of substitution with 6-, 8-, and 9-amino acid sequences, but surprisingly did not appear to be tolerant of a 7-amino acid loop. Roughly half of the enriched clones from the EL3-7 library contained substituted loops whose length deviated from the designed length of seven amino acids, despite the apparent absence of clones with incorrect loop lengths in sequences identified from the original library. We hypothesized that clones of incorrect loop lengths likely arose from infrequent impurities in the degenerate oligonucleotide or arose during the gene assembly process. To determine whether the trend for isolating clones of the incorrect lengths resulted from low library quality or from preference of the EETI scaffold, we constructed a second EL3-7 library using a highly purified degenerate oligonucleotide. After sorting the new EL3-7 library for trypsin binding as before, we again sequenced the enriched clones, but now found that they were all of the correct length. However, approximately 34% of the loop sequences contained an internal cysteine residue, potentially shortening the actual loop length from the intended seven amino acids to between four and six amino acids (Dataset S1). When these cysteine-containing loop sequences were disregarded, the remaining EL3-7 clones displayed sequence patterns in agreement with clones isolated from other EETI loop 3-substituted libraries, but had a higher frequency of proline residues.

### Covariance analysis of properly folded EETI loop 3-substituted clones

The enriched EL3-9 clones were chosen as a model group for further exploration of the EETI knottin scaffold because their trypsin-binding affinities were closest to that of EETIwt (Figure 2C). We performed covariance analysis on the substituted loop sequences of the enriched EL3-9 clones to determine whether the amino acid preferences at one loop position influenced the amino acid preferences at a second loop position. A comprehensive set of commonly used scoring functions [34] containing the following covariance algorithms was employed: Observed Minus Expected Squared (OMES) [35], Mutual Information (MI) [36], Statistical Coupling Analysis (SCA) [37], McLachlan Based Substitution Correlation (McBASC) [38],[39], and Explicit Likelihood of Subset Co-variation (ELSC) [40]. To determine background levels of covariance scores for each algorithm, we calculated the mean covariance and standard deviation across all positional pairs in the substituted loops of sequences from the unsorted EL3-9 library (Dataset S2). We then used the resulting background values to convert the covariance scores for positional pairs in enriched EL3-9 clones (Dataset S3) to standardized scores (z-scores).

The MI scoring function failed to give scores detectable over background noise, as the maximum z-score returned by this algorithm for any covarying pair was less than 0.1. The SCA algorithm identified the most covarying pairs. However, SCA-identified covarying pairs mainly contained highly conserved loop positions (i.e. positions 8 & 9), inhibiting the identification of correlated amino acids within coupled positions. For this reason, covarying pairs identified by SCA were discarded. The ELSC scoring function identified only a single covarying pair; this pair was redundant with results from the OMES algorithm. Therefore, only covariance scores calculated using the OMES and McBASC algorithms were considered for further analysis. Setting the covariance threshold to pairs with a standardized score greater than 2 (indicating a score two standard deviations above the background mean, significance threshold p<0.025) identified four covarying pairs of loop positions: positions 1 and 7, positions 2 and 4, positions 2 and 9, and positions 5 and 6 (Dataset S3). However, there was minimal agreement in the results from the three covariance algorithms used. Of the four covarying pairs, only one (positions 2 and 4) was predicted by more than one algorithm.

**Figure 6:**
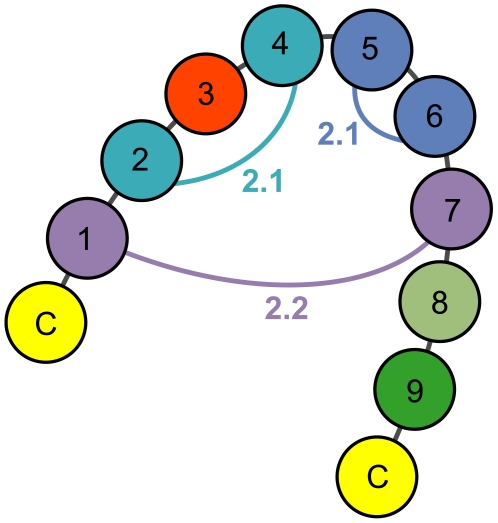
Covarying loop positions in EETI loop 3-substituted clones. Coupled loop positions in enriched EL3-9 clones are shown linked with their respective z-scores. Covariance patterns and correlated pairs of amino acids at each of the coupled positions were used to predict sequences of EETI loop 3 variants that adopt the knottin fold. For purposes of generating predictions, position three was set to asparagine or threonine, and positions eight and nine were set to glycine and tyrosine, respectively.

To identify predictive residues at these coupled positions, we manually analyzed each covarying position to uncover frequently occurring correlated amino acid pairs. Our analyses revealed multiple pairs of correlated amino acids (Table 1) at three of the four covarying loop positions (Figure 6). While several of the correlated amino acids were mutually predictive, other pairs displayed uni-directional predictivities. Due to the high level of conservation at loop position 9, we were unable to find correlated pairs of amino acids at positions 2 and 9 whose paired frequencies differed significantly from their occurrence rates in the overall population, so this covarying pair was excluded from further analysis.

**Table 1:** Common correlated amino acid pairs occurring at covarying loop positions.

Position i	Position j	Correlated Amino Acid Pairs (i, j)
1	7	(N⇔T), (N⇔R), (R⇔L), (V⇔L), (V⇒Y)
2	4	(R⇔R), (K⇔T), (T⇔N), (N⇐N), (P⇐N)
5	6	(R⇔R), (R⇒H), (N⇒S), (T⇐G), (H⇐G), (K⇐T), (G⇐T)

Directionality of arrows denotes amino acid predictivity.

### Prediction of tolerated amino acid sequences for EETI loop 3-substituted clones

We next used the results of our covariance analysis to predict sequences of nine-amino acid peptides that could be substituted into loop 3 of EETI without disrupting the knottin fold. Since loop positions three, eight, and nine were not predicted by covariance analysis, we constrained these residues using other observed sequence patterns. EETI loop 3 positions eight and nine were fixed to a glycine-tyrosine doublet because this combination of residues was present at the final two loop positions in 52% of enriched EL3-9 clones. EETI loop 3 position three was set to either asparagine or threonine, since these two amino acids frequently occurred in sequences containing a glycine-tyrosine doublet. We constrained the remaining loop positions with the identified covariance patterns and correlated pairs of amino acids (Figure 6 and Table 1). By exhaustively combining all possible pairs of covarying residues, we predicted a total of 420 tolerated loop sequences for substitution into EETI loop 3 (Dataset S4). Although the predicted sequences were generated by combining pairs of amino acids observed in the enriched EL3-9 library, none of the predicted sequences were identical to any of the enriched EL3-9 clones.

**Figure 7:**
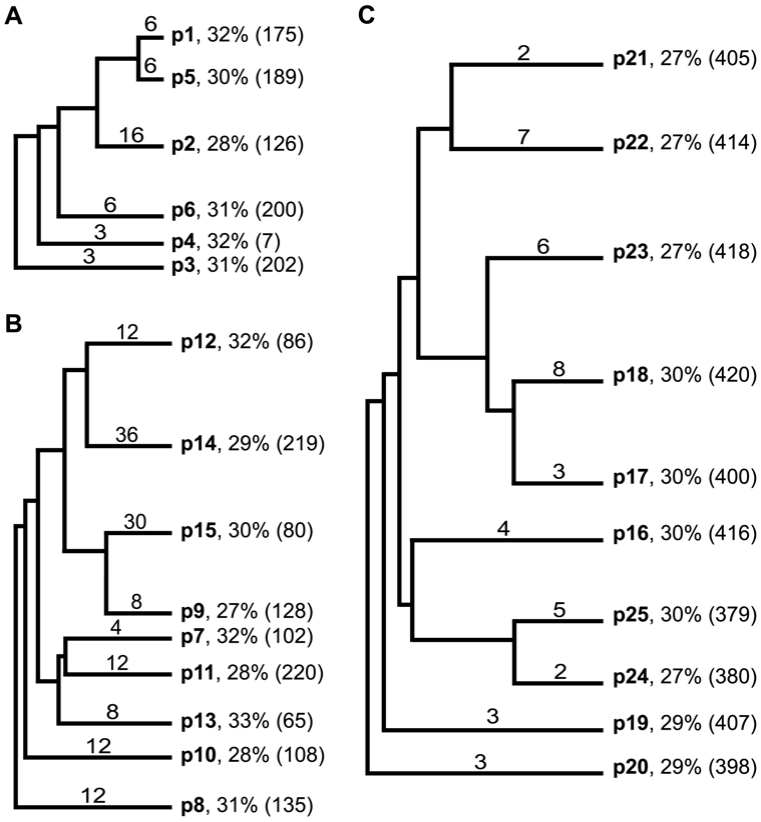
Cladograms of predicted EETI loop 3 clones tested for their abilities to bind trypsin. Select predicted EETI loop 3 clones tested for binding to fluorescently-labeled trypsin are shown in their relative groups: (A) clones containing four or more common motifs, (B) clones containing three common motifs, and (C) clones least similar to those recovered from the enriched EL3-9 library. Names of the predicted clones are shown in bold. Numbers above the branch lines denote the number of other predicted clones in the same cluster as the selected clone. Percentages denote the average homology of the loop sequence of the predicted clone to those of the pool of enriched EL3-9 clones. The similarity rankings of each clone to the pool of enriched EL3-9 trypsin-binding clones, based on a modified BLOSUM62 substitution matrix, are shown in parentheses (where 1 is most similar and 420 is least similar).

We then ranked our predicted peptide sequences according to the number of common motifs each shared with the loop 3 sequences of enriched clones from the EL3-9 library (Table S1). Here, we define a common motif as a discontinuous three-amino acid sequence pattern uncovered from analysis of enriched EL3-9 clones, not including the XXN/TXXXXGY motifs used to generate the predicted sequences. Because the common motifs used for ranking the predicted sequences all contained a C-terminal tyrosine or glycine-tyrosine pair, inclusion of these motifs allowed us to consider potential sequence preferences associated with the C-terminal positions of loop 3 whose conservation levels were too high to be detected by covariance analysis. We hypothesized that predicted peptides containing the greatest number of common motifs would be the least likely to disrupt the EETI fold. We found that 40 of the 420 predicted sequences contained four or more common motifs (Dataset S5) and 134 of the sequences contained three common motifs (Dataset S6). We aligned and grouped the 174 motif-filtered predicted sequences using ClustalW [41] and then chose a representative clone at random from each of the resulting fifteen subgroups to be tested for its ability to bind trypsin (Figure 7A and B). In addition to ranking our predicted clones by incorporation of common motifs, we used a scoring function based on a modified BLOSUM62 amino acid substitution matrix [42] to rank the predictions according to their similarities to clones isolated from the enriched EL3-9 library. To test the lower limits of our predictive capability, we used ClustalW to align predicted clones whose similarity scores were in the lowest 10% and selected the clone with the lowest similarity score from each subgroup to be tested for its trypsin-binding ability (Figure 7C). Finally, for comparison, we used the ExPASy RandSeq tool to generate random, nine-amino acid peptides with frequencies typical of NNS codons for substitution into EETI loop 3.

**Figure 8:**
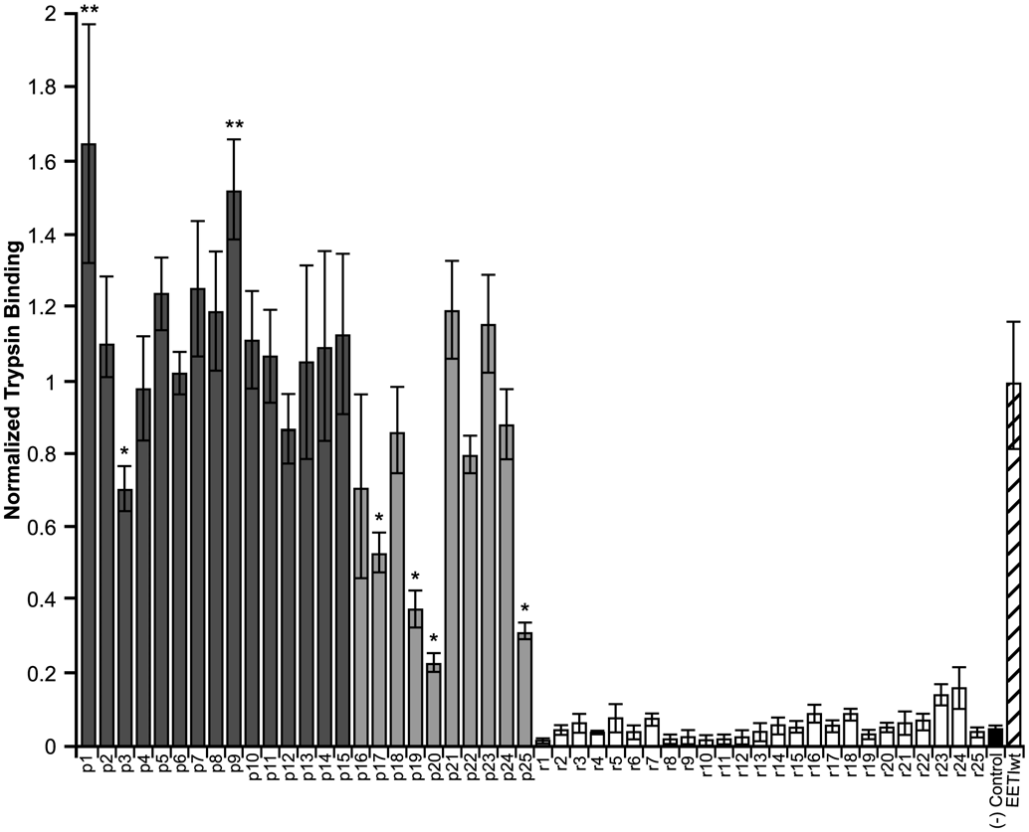
Trypsin-binding levels of EETI loop 3 predicted and randomly-generated clones. Motif-filtered clones (dark grey), least-similar clones (light grey), randomly generated clones (white), negative control (potato carboxypeptidase inhibitor knottin, black), and EETIwt (striped) were individually displayed on the surface of yeast and analyzed by flow cytometry. Predicted clones are preceded with a ‘p’ while randomly-generated clones are preceded with an ‘r.’ Protein expression levels were quantified by immunofluorescence staining of the cMyc epitope tag. Retention of the knottin fold was determined by binding of fluorescently-labeled trypsin (25 nM). Trypsin binding levels were adjusted to account for differences in protein expression levels and then normalized to the trypsin-binding level of EETIwt. Trypsin-binding experiments were performed in triplicate and error bars denote standard deviations. Predicted clones showing statistically significant differences in trypsin binding levels compared to EETIwt are marked with an asterisk (*) or a double asterisk (**) to indicate lower and higher binding levels, respectively.

We prepared twenty-five yeast display plasmids containing representative EETI loop 3-substituted clones that were predicted to be properly folded, and hence bind trypsin: fifteen motif-filtered sequences and ten least-similar sequences (Table S2). In addition, we also constructed twenty-five clones where EETI loop 3 was replaced by a randomly-generated nine-amino acid sequence (Table S2). Plasmids containing DNA encoding for predicted and randomly-generated clones were transformed into yeast and induced for expression on the yeast cell surface as described above. Next, we analyzed the predicted and randomly-generated EETI loop 3 clones by dual-color flow cytometry for yeast cell surface expression and binding to fluorescent trypsin, indicating retention of the knottin fold (Figure 8). Remarkably, we found that all motif-filtered predicted clones bound trypsin. Of the motif-filtered predicted clones, two (p1 and p9) showed trypsin binding levels roughly 1.5-fold higher than that of EETIwt and one (p3) showed trypsin binding levels approximately 70% that of EETIwt; these differences in binding were statistically significant, as determined by single-factor ANOVA (p<0.05). The remaining 12 motif-filtered predicted clones bound trypsin at levels comparable to that of EETIwt. Overall, the least-similar predicted clones also bound trypsin, but not as well as the motif-filtered clones, as expected. The small differences in trypsin binding levels of six of the least-similar predicted clones as compared to EETIwt were deemed statistically insignificant, based on single-factor ANOVA (p<0.05). Although two of the least-similar predicted clones (p20 and p25) bound trypsin at levels approximately 30% that of EETIwt, they still bound trypsin at levels that were statistically significant over the highest-binding randomly-generated clone, as determined by single-factor ANOVA (p<0.02). The majority of randomly-generated clones showed trypsin binding levels comparable to that of a negative control, the potato carboxypeptidase inhibitor knottin, which does not bind trypsin (<5% of the wild-type EETI trypsin binding level). Even the best randomly-generated clone only bound trypsin at 16% of the wild-type EETI trypsin binding level. The successful prediction of nine-amino acid sequences that can be substituted into EETI loop 3 without disrupting the knottin fold highlights the potential of this combinatorial approach for rational protein engineering applications.

## Discussion

The trypsin binding loop of EETI (loop 1) has previously been engineered for novel binding functionalities [15], [27]–[29],[43]. Although prior studies suggested that EETI loops 2 and 3 might be amenable to mutagenesis [24],[26], the potential for using these loops to confer stability or new recognition properties has not been previously investigated. In this study, we surveyed the tolerated sequence and loop length diversity of the EETI knottin to assess its utility as a scaffold for protein engineering applications. We displayed EETI loop-substituted variants on the surface of yeast and determined whether they retained the knottin fold by assaying their ability to bind trypsin. In the process, we developed a new method for understanding tolerated diversity based on isolating properly folded protein variants from highly diverse libraries, followed by comprehensive sequence analysis of the isolated clones. EETI was chosen as an optimal knottin scaffold for this study for a number of reasons. First, EETI has two solvent-exposed loops that are not involved in trypsin binding and do not have steric or electrostatic interactions with the trypsin-binding loop, making them well-suited for loop substitution [24]. Second, trypsin binding can be used to assess proper protein folding since removal of any of the three disulfide bonds in EETI (e.g. by cysteine to serine mutations) prevents formation of the cystine-knot and abolishes trypsin binding [26]. Third, the crystal structures of EETI variants previously selected for binding to trypsin depict wild-type-like knottin structures [24]. These studies provide significant support that only properly folded EETI variants will retain trypsin-binding capabilities, while variants that deviate from the knottin fold will be unable to bind trypsin. Taken together, the interaction between the N-terminal loop of EETI and trypsin provided a conformational handle for probing the structural effects of extended mutations on the remaining two EETI loops. Moreover, EETI has already been shown to be amenable to yeast surface display [28], as well as other directed evolution platforms including *Escherichia coli* cell surface display [26],[27], and mRNA display [44].

Yeast surface display has proven to be a robust platform for screening combinatorial libraries of proteins with disulfide bonds and complex folds, including antibodies [45], cell surface receptors [46], growth factors [47], and knottins [22],[28]. Additionally, yeast surface display allows for quantitative library screening using FACS, which enabled knottin protein expression levels to be correlated with trypsin binding levels with single-cell resolution. Such normalization allowed us to isolate clones that possessed the highest fraction of properly folded knottins over clones that displayed a large amount of misfolded proteins with only a small trypsin-binding subset. The quality control mechanisms of the yeast secretory pathway should prevent misfolded or incompletely folded proteins from being displayed on the cell surface [48],[49]. However, others have reported limitations in the ability of the yeast quality control system to differentiate between properly folded and unfolded variants of proteins with high thermal stabilities [50]. Therefore, it is possible that EETI loop-substituted clones that were expressed on yeast but did not bind trypsin were in an improperly folded state, such as the EETI two-disulfide intermediate [25]. Such clones may be sufficiently stable to escape the quality control machinery of the yeast secretory pathway. Alternatively, it is possible that some of the loop-substituted sequences introduced structural perturbations that were propagated along the polypeptide backbone, preventing the proper interaction of EETI loop 1 with trypsin despite retention of the native topology of the knottin fold.

We designed EETI libraries where loop 2 or loop 3 was substituted with randomized sequences to determine whether the knottin fold was affected by sequence modifications. In addition, we simultaneously tested the effects of varying loop size on the knottin fold by replacing the loops with randomized peptides of different lengths. For these studies, we explored only loop lengths longer than those naturally occurring in the EETI knottin. Our choice of loop lengths was influenced by several factors: 1) the utility of the knottin scaffold for protein engineering applications relies on the ability to evolve novel molecular recognition epitopes, and high-affinity interactions typically require binding interfaces larger than the native loop lengths of EETI (4–5 residues), and 2) although loop lengths of 7–17 amino acids have previously been grafted in place of the EETI trypsin binding loop [27],[29], loops of longer lengths are less structurally constrained and the entropic binding advantage of presenting the loop on a scaffold is mitigated. Hence, we limited the loop lengths tested to between six and nine amino acids.

In some previous examples of knottin engineering, a sequence of interest was grafted into a constrained knottin loop on the premise that the new sequence would be structurally tolerated [17],[29],[43]. This assumption of tolerance was based on the lack of sequence homology that exists between proteins that share the knottin fold. We found a moderate degree of functional diversity amongst properly folded EETI loop-substituted clones (0.01%–0.9%, corresponding to enriched populations of 10^5^–10^7^), but not complete tolerance. In addition, sequences of properly folded EETI loop-substituted clones were largely composed of residues that would likely maintain the local secondary structure of the native EETI knottin fold. In wild-type EETI, loop 2 (KQDSD) composes a loop and short alpha helix that lies directly after the first beta sheet. It was therefore not surprising that the preferred amino acid residues found to populate loop 2 in properly folded EETI mutants were glycine, proline, and serine; these residues typically disrupt secondary structures and would therefore be conducive to the formation of a loop. Amino acids that preferentially adopt alpha helical structures were the next most abundant: alanine, leucine, and arginine collectively accounted for 35% of the remaining residues in properly folded EETI loop 2-substituted clones. The wild-type EETI loop 3 sequence (GPNGF) lies within a loop region and the beginning of the third anti-parallel beta sheet in the native structure. Similarly, we observed that properly folded EETI loop 3-substituted clones contained amino acids at the beginning of their loop sequences that favor the formation of a loop, such as asparagine, glycine, proline, and serine. The preferred glycine-tyrosine doublet found at the C-terminus of EETI loop 3 clones enriched for trypsin binding is similar to the glycine-phenylalanine sequence at the same location in the native loop; both doublets would be effective initiators of a beta sheet secondary structure. It is interesting to note that while histidine, threonine, tryptophan, and tyrosine are not present in the wild-type EETI sequence, each appeared in the sequences of loop-substituted clones, indicating there is not an intrinsic structural bias against these amino acids.

While the observed sequence trends were found to be specific to the location of the substituted loop, they were largely independent of loop length. The overall tolerance of the EETI knottin fold to a variety of substitution lengths in loop 2 and loop 3 is an important attribute for its potential as a molecular scaffold. Interestingly, the high tolerance of variations in loop length was greater than what might be anticipated from analysis of natural loop lengths across members of the knottin family. Over 40% of naturally-occurring knottins have a loop 2 length of five amino acids, while less than 10% and less than 5% have loop 2 lengths of seven and nine amino acids, respectively [51]. There is an intriguing parallel between observed loop 3 lengths in naturally-occurring knottins and the tolerated loop lengths we observed. The most common loop 3 lengths of knottins are four (more than 25%), six (nearly 20%), or ten (nearly 15%) amino acids, while loop lengths of seven or eight amino acids each occur in less than 5% of the knottin family [51]. This is congruous to our findings here: a loop 3 substitution length of seven amino acids was found unfavorable and EETI loop 3-substituted clones with loop lengths of six or nine amino acids were enriched to high levels of trypsin binding after FACS.

We used sequence covariance analysis to identify inter-residue couplings at positions along the length of properly folded EETI loop 3-substituted clones. Covariance analysis can be applied to proteins to highlight structurally or functionally important residues and has previously been employed for a myriad of purposes, including revealing inter-residue contacts within protein structures [38],[52], protein folding pathways [53], energetic coupling pathways [37], communication pathways of allosteric proteins [35], and correlated mutations involved in drug-resistance [54]. In addition, covariance analysis has been used to predict protein structure [39], predict protein-protein interactions [55], and guide protein docking experiments [56]. Although we performed covariance analysis on the loop 3-substituted sequences of trypsin-binding EETI mutants using several different algorithms [34], only the results from the OMES [35] and McBASC [38],[39] scoring functions were used to generate sequence predictions. The lack of agreement among results calculated with the various covariance algorithms likely stems from differences in their sensitivities to amino acid conservation levels. Indeed, the OMES and McBASC scoring functions have been reported to have similar sensitivities to background levels of conservation, resulting in similar scoring performances for covariance analysis on sets of Pfam protein families [34]. The sensitivity of our covariance and correlated mutation analyses was limited by the quantity of sequencing data available (52 and 56 sequences for the enriched and starting EL3-9 libraries, respectively). Because of the small sample size of our sequencing data, we were able to detect only the strongest trends over background noise; we anticipate that there exist other, more subtle connectivities and less prevalent correlated pairs of amino acid residues within the substituted loop regions that would be revealed with a larger data set. However, it is notable that even a small dataset was able to predict hundreds of functionally tolerated sequences.

We applied the results of our covariance analysis to predict nine-amino acid sequences for substitution into EETI loop 3 that lead to proper folding of the protein. The exhaustive combination of all possible correlated mutations at three covarying positional pairs and three constant loop positions afforded 420 predicted clones. Although the rational design of a tolerated loop sequence was aided by the stability of the knottin fold, there are 15 possible ways to form three disulfide bonds from six cysteine residues, and folding and oxidation must occur in coordination to result in a cystine-knot topology [7]. The task of designing sequences for substitution into EETI loop 3 was further complicated by the important role of this loop in the folding of the knottin. The residues of EETI loop 3 initiate the folding of the knottin structure by forming a beta-turn that is responsible for facilitating the association of the anti-parallel beta sheets prior to disulfide bond formation [23],[25]; furthermore, mutations to EETI loop 3 have been observed to result in misfolded by-products [26]. The results of our studies, in which EETI loop 3 clones that properly fold and bind to trypsin were predicted, demonstrate that covariance and correlated mutation analysis can be successfully used for rational protein engineering. Moreover, trends identified by sequence and covariance analyses provide guidelines for introducing diversity into knottin loop regions when minimal structural disruption is desired, for example, when performing directed evolution experiments.

Pál and colleagues previously used phage display and covariance analysis to design pacifastin protease inhibitors with altered binding specificities for trypsin [57]. In another study, Ranganathan and colleagues used covariance analysis to computationally predict artificial sequences of properly folded and functional WW domain proteins [58],[59]. These studies suggested that a combination of amino acid conservation and covariance analysis is necessary and sufficient to inform successful protein design. This finding is corroborated in our studies; we used both conserved amino acids and covariance analysis with correlated mutations to design EETI loop 3 variants that adopt the cystine-knot topology. While both of these previous studies were based on genetic information from naturally-occurring proteins, our study used sequences isolated from a naive library of loop-randomized protein variants. This distinction extends the applicability of covariance analysis for protein design to include sequences that differ significantly from their naturally-occurring counterparts not only in amino acid composition, but also in sequence length. Indeed, our approach successfully predicted artificial EETI-based knottin proteins with substituted loop sequences 1.5–2 fold longer than those found in the most closely related, naturally-occurring knottin family members. Further, the covariance analysis of clones isolated from a naive loop-substituted library permits the exploration of greater diversity than analysis of a family of naturally-occurring or naturally-derived proteins, potentially resulting in predicted artificial protein sequences with greater diversity. Simultaneous maintenance of this diversity and compliance with minimal structural requirements is essential for engineering novel characteristics (e.g. molecular recognition) into scaffold proteins while ensuring that the protein structure is not compromised.

Upon analysis of the knottin database [51],[60], we found loop sequences in naturally-occurring knottins that were homologous to those of clones from the enriched EL3-9 library. Such homologous sequences were found in several serine protease inhibitor knottins, which are functionally related to EETI. Surprisingly, we also found natural loop sequences that shared at least 50% homology with those of enriched EL3-9 clones from knottin families functionally unrelated to EETI, including conotoxin, spider toxin, plant toxin, and scorpion toxin (Table S3). Further, the C-terminal glycine-tyrosine doublet present in enriched EL3-9 clones was also found in the sequences of many naturally-occurring knottins belonging to the fungi, insect antimicrobial, plant antimicrobial, and trematoda families (Table S3). The observation that sequences similar to those of our enriched EETI loop 3-substituted clones exist in the loops of naturally-occurring knottins that are functionally unrelated to EETI suggests that the covariance patterns we identified here may be useful for engineering other members of the knottin family.

In summary, this work has shown the feasibility of using yeast-displayed libraries of EETI loop-substituted proteins to investigate the structural tolerances of a knottin fold in a predictive manner. Since combinatorial library sizes are limited by host transformation efficiencies and rational design thus far has been met with limited success, a set of guidelines for biasing starting libraries is valuable. This is especially true for the knottin scaffold, whose advantageous characteristics (e.g. high thermal stability and resistance to proteolysis) are dependent on the correct disulfide-bonded topologies. Finally, this work demonstrates the potential of using directed evolution platforms in combination with covariance analysis to guide future efforts in engineering functional proteins whose sequences differ from their naturally-occurring counterparts not only in amino acid composition, but also in sequence length.

## Materials and Methods

### Media and reagents

YPD media contained 20 g/L dextrose, 20 g/L peptone, and 10 g/L yeast extract. Selective SD-CAA media contained 20 g/L dextrose, 6.7 g/L yeast nitrogen base without amino acids, 5.4 g/L Na_2_HPO_4_, 8.6 g/L NaH_2_PO_4_⋅H_2_O, and 5 g/L Bacto casamino acids. Selective SD-CAA plates were of the same composition as the liquid media except with the addition of 182 g/L sorbitol and 15 g/L agar. SG-CAA media was identical to the SD-CAA media except dextrose was replaced with galactose. PBSA was composed of phosphate buffered saline containing 1 g/L bovine serum albumin. Lyophilized trypsin was purchased from Sigma Aldrich and fluorescently labeled with Alexa Fluor 488 tetrafluorophenyl ester (Invitrogen). Mouse anti-cMyc antibody was purchased from Covance and goat anti-mouse antibody conjugated to R-phycoerythrin was purchased from Sigma Aldrich.

### Construction of EETI loop-substituted libraries

EETI loop-substituted knottin libraries were constructed by overlap extension PCR using KOD polymerase (Novagen) in the presence of 1 M betaine and 3% dimethylsulfoxide. Oligonucleotides were designed using yeast-optimized codons, with randomized loop positions encoded by the degenerate NNS codon. Assembled PCR products were amplified with Pfx50 polymerase (Invitrogen) using forward and reverse oligonucleotides with 45 bp homology upstream or downstream of the *Nhe*I and *Bam*HI restriction sites, respectively, in the pCT yeast display vector [31]. The pCT yeast display vector was digested with *Nhe*I and *Bam*HI restriction enzymes (New England Biolabs) and treated with calf intestinal phosphatase (New England Biolabs). Amplified PCR products of the correct lengths and linearized pCT vector backbone were separated by electrophoresis on a 2% agarose gel and purified using a QIAquick Gel Extraction Kit (Qiagen). EETI loop-substituted DNA inserts (10–15 µg) and linearized pCT vector (1–1.5 µg) were transformed into EBY100 yeast [30] by electroporation [31] at a ratio of 10∶1. After electroporation, yeast were allowed to recover in YPD at 30°C for 1 h with shaking prior to transfer to selective growth media. Libraries were propagated in selective SD-CAA media and induced for protein expression in SG-CAA media at 30°C. Library sizes were estimated by plating serial dilutions onto selective SD-CAA agar plates and colony counting.

### Screening of EETI-loop substituted libraries

Four rounds of FACS were performed on each EETI loop-substituted library to obtain an enriched pool of trypsin-binding clones. Libraries of yeast clones induced for protein expression were suspended in PBSA with mouse anti-cMyc monoclonal antibody (1∶50 dilution) and incubated for 1 h at room temperature. Yeast were pelleted by centrifugation at 4,000 rpm for 5 min, the supernatant was aspirated, and cells were washed with ice-cold PBSA. Washed yeast libraries were resuspended in PBSA with goat anti-mouse R-phycoerythrin secondary antibody (1∶25 dilution) and Alexa 488-labeled trypsin and incubated on ice in the dark for 30 min. Yeast libraries were washed as before and screened by dual-color FACS for mutants that were both displayed on the yeast surface and bound to trypsin using a Becton Dickinson FACSVantage SE instrument (Stanford FACS Core Facility) and CellQuest software (Becton Dickinson). Collected library clones were propagated in SD-CAA media with penicillin-streptomycin (400 µg/mL), induced for protein expression in SG-CAA media and subjected to three additional rounds of sorting. For the first round of sorting, approximately 2×10^7^ yeast were sorted from each library, and at least 10 times the number of collected yeast were sorted in each subsequent round to decrease the probability of losing unique clones. Sort stringency was increased by gradually decreasing the concentration of Alexa488-labeled trypsin from 200 nM in the first round to 25 nM in the fourth round.

### Sequence analysis of EETI loop-substituted clones

Plasmid DNA from the original and FACS-enriched libraries were recovered from the yeast using a Zymoprep kit (Zymo Research) and then transformed into XL1-blue supercompetent *E. coli* (Strategene). Transformed *E. coli* were incubated in SOC media (Invitrogen) at 37°C for 1 h with shaking before plating on LB agar plates with ampicillin (100 µg/mL). At least 50 unique clones from each of the original and enriched EETI loop-substituted libraries were recovered and sequenced. Elim Biopharmaceuticals (Hayward, CA) and MCLAB (South San Francisco, CA) performed plasmid sequencing services. Only clones without truncations and with loops of the correct target length were included in the analysis below. Library sequences were analyzed using programs available on the RELIC bioinformatics server [61]. The AAFREQ program was used to calculate overall and positional frequencies of each amino acid residue in the substituted loop regions. POPDIV [32] was used to estimate the diversity within the original and sorted EETI loop-substituted library populations. The DIVAA program [33] was used to quantify the tolerated diversity at each position of the randomized loops of EETI loop-substituted library clones, both in the original and enriched libraries. The MOTIF1 and MOTIF2 programs [61] were used to identify continuous and discontinuous motifs, respectively, within the loop regions of EL3-9 clones isolated from the fourth round of FACS.

### Covariance analysis of EETI loop 3-substituted clones

Substituted loop sequences from the original and enriched EL3-9 libraries were aligned using ClustalW2 [41] with default parameters, except the penalty for opening a gap was set to 100 instead of 15. Covariance analysis was performed on the aligned sequences to identify coupled loop positions. The analysis was conducted using the OMES, ELSC, MI, SCA, and McBASC algorithms as previously described [34]. Covariance analysis with each of the algorithms was first performed on sequences from the original, unsorted EL3-9 library, which served as a negative control for covariance since it was designed to have randomized loops. To quantify the average background covariance score inherent to each method, we calculated the average scores and standard deviations obtained using each of the algorithms across all positional loop pairs in the unsorted EL3-9 library.

Covariance analysis was then performed on the aligned sequences from the enriched EL3-9 library. The resulting covariance scores for each positional pair were converted into z-scores using the average and standard deviation values obtained for the original library with each corresponding algorithm. Positional pairs with z-scores greater than or equal to 2 were individually analyzed for common correlated mutations in amino acid residues. Those positional amino acid pairs (i, j) whose frequency was at least 50% greater as a matched pair than their individual frequencies in the sorted population were used to predict tolerated loop sequences. To minimize error introduced by small sample sizes, only those residues that populated the predictor position in at least 10% of the sequences were considered for analysis. Further, predicted amino acids were only considered if they occurred at least twice in the general population.

### Prediction of tolerated nine-amino acid sequences for substitution into EETI loop 3

Predicted peptide sequences were generated for substitution into EETI loop 3 by combining the trends uncovered in the sequence and covariance analyses of the enriched EL3-9 trypsin-binding clones. The third position of EETI loop 3 was set to either asparagine or threonine based on frequently-occurring motifs and positions 8 and 9 were set to the observed glycine-tyrosine consensus sequence. The remaining positions of EETI loop 3 were predicted according to the results from covariance analysis. All possible combinations of correlated amino acid pairs were used to generate 420 clones that we predicted would retain binding to trypsin. The predicted clones were then filtered based on their inclusion of common motifs observed in the EL3-9 sorted library. Predicted clones whose loop 3 sequences contained 3 or 4 common motifs were aligned using ClustalW2 [41] and 15 clones representative of the predicted sequence space were chosen at random for testing of their trypsin-binding abilities. Additionally, the 420 predicted clones were ranked according to their similarities (calculated based on a modified BLOSUM62 matrix) to clones from the enriched EL3-9 library using the FASTAskan program [42] from the RELIC bioinformatics server [61]. Predicted clones whose similarity scores were in the lowest 10% were aligned with ClustalW2 and the 10 lowest ranked clones representative of the sequence space were selected for testing. Additionally, we used the RandSeq tool (http://ca.expasy.org/tools/randseq.html) from the ExPASy server to generate 25 EETI clones whose loop 3 sequences were replaced with randomized, nine-amino acid sequences. For this purpose, amino acid compositions were set according to the frequencies expected from degenerate NNS codons.

### Testing predicted EETI loop 3 clones for trypsin binding

The 25 predicted EETI loop 3 clones and the 25 randomly-generated EETI loop 3 clones were constructed by PCR with overlapping primers, digested with *Nhe*I and *Bam*HI restriction enzymes, and ligated into linearized pCT vector with T4 DNA ligase (New England Biolabs). Ligated pCT-EETI loop 3 predicted and randomly-generated plasmids were transformed into XL1-blue supercompetent *E. coli* (Stratagene) for plasmid miniprep and sequencing (MCLAB). Clones of the correct sequences were transformed into *S. cerevisiae* strain EBY100 yeast by electroporation and grown on selective SD-CAA agar plates. For each EETI clone, three individual yeast colonies were selected from the corresponding transformation plate, propagated in selective SD-CAA media, and induced for protein expression in SG-CAA media at 30°C. Thus, triplicate samples of yeast-displayed EETI loop 3 clones were analyzed by dual-color flow cytometry for protein expression and trypsin binding at 25 nM as described above.

## Supporting Information

Table S1 Common motifs found in the randomized loops of enriched EL3-9 clones.
(0.04 MB DOC)

Table S2 Sequences of predicted and randomly-generated clones individually tested for binding to trypsin.
(0.07 MB DOC)

Table S3 Representative naturally-occurring knottins with loop sequences similar to those of enriched EL3-9 clones. C-terminal glycine-tyrosine doublets are shown in bold and the corresponding loop sequences are underlined. Clones whose underlined loop sequence is at least 50% homologous to the loop sequence of a clone from the enriched EL3-9 library are italicized and bolded. Sequences are listed according to their sub-family groupings in the KNOTTIN database (http://knottin.cbs.cnrs.fr/Knottins.php) and are named according to their UniprotKB/Swiss-Prot accession numbers.
(0.07 MB DOC)

Dataset S1 Recovered sequences of randomized loops from the unsorted and enriched EETI loop-substituted libraries.
(0.37 MB DOC)

Dataset S2 Raw covariance scores for the unsorted EL3-9 library calculated using the OMES, SCA, ELSC, MI, and McBASC scoring functions.
(0.09 MB DOC)

Dataset S3 Raw covariance scores for the enriched EL3-9 library calculated using the OMES, SCA, ELSC, MI, and McBASC scoring functions. Scores shown in bold are those whose corresponding z-scores are greater than 2.
(0.10 MB DOC)

Dataset S4 Predicted nine-amino acid sequences for substitution into EETI loop 3.
(0.07 MB DOC)

Dataset S5 Multiple sequence alignment of predicted EETI loop 3 sequences containing four or more common motifs. Numbers correspond to clone numbers as assigned in Dataset S4. The alignment was generated with ClustalW v.2.0.10.
(0.05 MB DOC)

Dataset S6 Multiple sequence alignment of predicted EETI loop 3 sequences containing three common motifs. Numbers correspond to clone numbers as assigned in Dataset S4. The alignment was generated with ClustalW v.2.0.10.
(0.06 MB DOC)

## References

[1] Silverman J, Liu Q, Bakker A, To W, Duguay A (2005). Multivalent avimer proteins evolved by exon shuffling of a family of human receptor domains.. Nat Biotechnol.

[2] Parker MH, Chen Y, Danehy F, Dufu K, Ekstrom J (2005). Antibody mimics based on human fibronectin type three domain engineered for thermostability and high-affinity binding to vascular endothelial growth factor receptor two.. Protein Eng Des Sel.

[3] Shusta EV, Holler PD, Kieke MC, Kranz DM, Wittrup KD (2000). Directed evolution of a stable scaffold for T-cell receptor engineering.. Nat Biotechnol.

[4] Hey T, Fiedler E, Rudolph R, Fiedler M (2005). Artificial, non-antibody binding proteins for pharmaceutical and industrial applications.. Trends Biotechnol.

[5] Skerra A (2007). Alternative non-antibody scaffolds for molecular recognition.. Curr Opin Biotechnol.

[6] Binz HK, Amstutz P, Pluckthun A (2005). Engineering novel binding proteins from nonimmunoglobulin domains.. Nat Biotechnol.

[7] Kolmar H (2008). Alternative binding proteins: biological activity and therapeutic potential of cystine-knot miniproteins.. Febs J.

[8] Andersson M, Ronnmark J, Arestrom I, Nygren PA, Ahlborg N (2003). Inclusion of a non-immunoglobulin binding protein in two-site ELISA for quantification of human serum proteins without interference by heterophilic serum antibodies.. J Immunol Methods.

[9] Reina J, Lacroix E, Hobson SD, Fernandez-Ballester G, Rybin V (2002). Computer-aided design of a PDZ domain to recognize new target sequences.. Nat Struct Biol.

[10] Gill DS, Damle NK (2006). Biopharmaceutical drug discovery using novel protein scaffolds.. Curr Opin Biotechnol.

[11] Colas P, Cohen B, Jessen T, Grishina I, McCoy J (1996). Genetic selection of peptide aptamers that recognize and inhibit cyclin-dependent kinase 2.. Nature.

[12] Xu L, Aha P, Gu K, Kuimelis RG, Kurz M (2002). Directed evolution of high-affinity antibody mimics using mRNA display.. Chem Biol.

[13] Helms LR, Wetzel R (1995). Destabilizing loop swaps in the CDRs of an immunoglobulin VL domain.. Protein Sci.

[14] Nagi AD, Regan L (1997). An inverse correlation between loop length and stability in a four-helix-bundle protein.. Fold Des.

[15] Le Nguyen D, Heitz A, Chiche L, Castro B, Boigegrain RA (1990). Molecular recognition between serine proteases and new bioactive microproteins with a knotted structure.. Biochimie.

[16] Colgrave ML, Craik DJ (2004). Thermal, chemical, and enzymatic stability of the cyclotide kalata B1: the importance of the cyclic cystine knot.. Biochemistry.

[17] Hilpert K, Wessner H, Schneider-Mergener J, Welfle K, Misselwitz R (2003). Design and characterization of a hybrid miniprotein that specifically inhibits porcine pancreatic elastase.. J Biol Chem.

[18] Le-Nguyen D, Nalis D, Castro B (1989). Solid phase synthesis of a trypsin inhibitor isolated from the Cucurbitaceae Ecballium elaterium.. Int J Pept Protein Res.

[19] Chen XM, Qian YW, Chi CW, Gan KD, Zhang MF (1992). Chemical synthesis, molecular cloning, and expression of the gene coding for the Trichosanthes trypsin inhibitor–a squash family inhibitor.. J Biochem.

[20] Escoubas P, Bernard C, Lambeau G, Lazdunski M, Darbon H (2003). Recombinant production and solution structure of PcTx1, the specific peptide inhibitor of ASIC1a proton-gated cation channels.. Protein Sci.

[21] Ji W, Zhang X, Hu H, Chen J, Gao Y (2005). Expression and purification of Huwentoxin-I in baculovirus system.. Protein Expr Purif.

[22] Silverman AP, Levin AM, Lahti JL, Cochran JR (2009). Engineered cystine-knot peptides that bind alphavbeta3 integrin with antibody-like affinities.. J Mol Biol.

[23] Heitz A, Chiche L, Le-Nguyen D, Castro B (1989). 1H 2D NMR and distance geometry study of the folding of Ecballium elaterium trypsin inhibitor, a member of the squash inhibitors family.. Biochemistry.

[24] Kratzner R, Debreczeni JE, Pape T, Schneider TR, Wentzel A (2005). Structure of Ecballium elaterium trypsin inhibitor II (EETI-II): a rigid molecular scaffold.. Acta Crystallogr D Biol Crystallogr.

[25] Le-Nguyen D, Heitz A, Chiche L, el Hajji M, Castro B (1993). Characterization and 2D NMR study of the stable [9–21, 15–27] 2 disulfide intermediate in the folding of the 3 disulfide trypsin inhibitor EETI II.. Protein Sci.

[26] Wentzel A, Christmann A, Kratzner R, Kolmar H (1999). Sequence requirements of the GPNG beta-turn of the Ecballium elaterium trypsin inhibitor II explored by combinatorial library screening.. J Biol Chem.

[27] Christmann A, Walter K, Wentzel A, Kratzner R, Kolmar H (1999). The cystine knot of a squash-type protease inhibitor as a structural scaffold for Escherichia coli cell surface display of conformationally constrained peptides.. Protein Eng.

[28] Kimura RH, Levin AM, Cochran FV, Cochran JR (2009). Engineered cystine knot peptides that bind alphavbeta3, alphavbeta5, and alpha5beta1 integrins with low-nanomolar affinity..

[29] Reiss S, Sieber M, Oberle V, Wentzel A, Spangenberg P (2006). Inhibition of platelet aggregation by grafting RGD and KGD sequences on the structural scaffold of small disulfide-rich proteins.. Platelets.

[30] Boder ET, Wittrup KD (1997). Yeast surface display for screening combinatorial polypeptide libraries.. Nat Biotechnol.

[31] Chao G, Lau WL, Hackel BJ, Sazinsky SL, Lippow SM (2006). Isolating and engineering human antibodies using yeast surface display.. Nat Protoc.

[32] Makowski L, Soares A (2003). Estimating the diversity of peptide populations from limited sequence data.. Bioinformatics.

[33] Rodi DJ, Mandava S, Makowski L (2004). DIVAA: analysis of amino acid diversity in multiple aligned protein sequences.. Bioinformatics.

[34] Fodor AA, Aldrich RW (2004). Influence of conservation on calculations of amino acid covariance in multiple sequence alignments.. Proteins.

[35] Kass I, Horovitz A (2002). Mapping pathways of allosteric communication in GroEL by analysis of correlated mutations.. Proteins.

[36] Atchley WR, Wollenberg KR, Fitch WM, Terhalle W, Dress AW (2000). Correlations among amino acid sites in bHLH protein domains: an information theoretic analysis.. Mol Biol Evol.

[37] Lockless SW, Ranganathan R (1999). Evolutionarily conserved pathways of energetic connectivity in protein families.. Science.

[38] Gobel U, Sander C, Schneider R, Valencia A (1994). Correlated mutations and residue contacts in proteins.. Proteins.

[39] Olmea O, Rost B, Valencia A (1999). Effective use of sequence correlation and conservation in fold recognition.. J Mol Biol.

[40] Dekker JP, Fodor A, Aldrich RW, Yellen G (2004). A perturbation-based method for calculating explicit likelihood of evolutionary co-variance in multiple sequence alignments.. Bioinformatics.

[41] Larkin MA, Blackshields G, Brown NP, Chenna R, McGettigan PA (2007). Clustal W and Clustal X version 2.0.. Bioinformatics.

[42] Rodi DJ, Agoston GE, Manon R, Lapcevich R, Green SJ (2001). Identification of small molecule binding sites within proteins using phage display technology.. Comb Chem High Throughput Screen.

[43] Krause S, Schmoldt HU, Wentzel A, Ballmaier M, Friedrich K (2007). Grafting of thrombopoietin-mimetic peptides into cystine knot miniproteins yields high-affinity thrombopoietin antagonists and agonists.. Febs J.

[44] Baggio R, Burgstaller P, Hale SP, Putney AR, Lane M (2002). Identification of epitope-like consensus motifs using mRNA display.. J Mol Recognit.

[45] Kieke MC, Cho BK, Boder ET, Kranz DM, Wittrup KD (1997). Isolation of anti-T cell receptor scFv mutants by yeast surface display.. Protein Eng.

[46] Holler PD, Holman PO, Shusta EV, O'Herrin S, Wittrup KD (2000). In vitro evolution of a T cell receptor with high affinity for peptide/MHC.. Proc Natl Acad Sci U S A.

[47] Cochran JR, Kim YS, Lippow SM, Rao B, Wittrup KD (2006). Improved mutants from directed evolution are biased to orthologous substitutions.. Protein Eng Des Sel.

[48] Ellgaard L, Helenius A (2003). Quality control in the endoplasmic reticulum.. Nat Rev Mol Cell Biol.

[49] Kowalski JM, Parekh RN, Mao J, Wittrup KD (1998). Protein folding stability can determine the efficiency of escape from endoplasmic reticulum quality control.. J Biol Chem.

[50] Park S, Xu Y, Stowell XF, Gai F, Saven JG (2006). Limitations of yeast surface display in engineering proteins of high thermostability.. Protein Eng Des Sel.

[51] Gracy J, Le-Nguyen D, Gelly JC, Kaas Q, Heitz A (2008). KNOTTIN: the knottin or inhibitor cystine knot scaffold in 2007.. Nucleic Acids Res.

[52] Shindyalov IN, Kolchanov NA, Sander C (1994). Can three-dimensional contacts in protein structures be predicted by analysis of correlated mutations?. Protein Eng.

[53] Ortiz AR, Kolinski A, Skolnick J (1998). Fold assembly of small proteins using monte carlo simulations driven by restraints derived from multiple sequence alignments.. J Mol Biol.

[54] Hoffman NG, Schiffer CA, Swanstrom R (2003). Covariation of amino acid positions in HIV-1 protease.. Virology.

[55] Fariselli P, Olmea O, Valencia A, Casadio R (2001). Prediction of contact maps with neural networks and correlated mutations.. Protein Eng.

[56] Pazos F, Helmer-Citterich M, Ausiello G, Valencia A (1997). Correlated mutations contain information about protein-protein interaction.. J Mol Biol.

[57] Szenthe B, Patthy A, Gaspari Z, Kekesi AK, Graf L (2007). When the surface tells what lies beneath: combinatorial phage-display mutagenesis reveals complex networks of surface-core interactions in the pacifastin protease inhibitor family.. J Mol Biol.

[58] Russ WP, Lowery DM, Mishra P, Yaffe MB, Ranganathan R (2005). Natural-like function in artificial WW domains.. Nature.

[59] Socolich M, Lockless SW, Russ WP, Lee H, Gardner KH (2005). Evolutionary information for specifying a protein fold.. Nature.

[60] Gelly JC, Gracy J, Kaas Q, Le-Nguyen D, Heitz A (2004). The KNOTTIN website and database: a new information system dedicated to the knottin scaffold.. Nucleic Acids Res.

[61] Mandava S, Makowski L, Devarapalli S, Uzubell J, Rodi DJ (2004). RELIC–a bioinformatics server for combinatorial peptide analysis and identification of protein-ligand interaction sites.. Proteomics.

