# Supplementary material for: Interrogating and Predicting Tolerated Sequence Diversity in Protein Folds: Application to E. elaterium Trypsin Inhibitor-II Cystine-Knot Miniprotein
Source: PLoS Comput Biol. 2009 Sep 4;5(9):e1000499. doi: 10.1371/journal.pcbi.1000499 (PMC2725296; doi:10.1371/journal.pcbi.1000499)
Supplement: Table S1 — Common motifs found in the randomized loops of enriched EL3-9 clones. (0.04 MB DOC) [file pcbi.1000499.s001.doc]

**Table S1. Common motifs found in the randomized loops of enriched EL3-9 clones.**

| **Motif** | **Percent Occurrence** |
| --- | --- |
| nxxxxxxgy | 25.00 |
| nxgy | 11.54 |
| rxxxgy | 11.54 |
| vxxxxxxgy | 11.54 |
| kxxxxxgy | 9.62 |
| nxxxxgy | 9.62 |
| nxxxrxxxy | 9.62 |
| rxxgy | 9.62 |
| rxrxxxxy | 9.62 |
| txgy | 9.62 |
| txxxgy | 9.62 |
| txxxxgy | 9.62 |
| txxxxxgy | 9.62 |
