# Supplementary material for: Interrogating and Predicting Tolerated Sequence Diversity in Protein Folds: Application to E. elaterium Trypsin Inhibitor-II Cystine-Knot Miniprotein
Source: PLoS Comput Biol. 2009 Sep 4;5(9):e1000499. doi: 10.1371/journal.pcbi.1000499 (PMC2725296; doi:10.1371/journal.pcbi.1000499)
Supplement: Table S2 — Sequences of predicted and randomly-generated clones individually tested for binding to trypsin. (0.07 MB DOC) [file pcbi.1000499.s002.doc]

**Table S2. Sequences of predicted and randomly-generated clones individually tested for binding to trypsin.**

| **Predicted Clones: Motif-Filtered** | p1 | NRNRRRTGY | **Randomly-Generated Clones** | r1 | RYQMEVPAG |
| --- | --- | --- | --- | --- | --- |
|  | p2 | VRNRKTLGY |  | r2 | FLRLSPQYM |
|  | p3 | NRNRKTPGY |  | r3 | QPYNLVLGR |
|  | p4 | NKNTRRPGY |  | r4 | PVWLFAMQS |
|  | p5 | NRTRRHPGY |  | r5 | LRGQRKNAS |
|  | p6 | NRTRKTPGY |  | r6 | AKSFTRESM |
|  | p7 | NTNNRRRGY |  | r7 | RWTRSSYPK |
|  | p8 | NTNNKTTGY |  | r8 | ESGAMTNFL |
|  | p9 | RRNRKTLGY |  | r9 | WYGKETVRM |
|  | p10 | RTNNKTLGY |  | r10 | GNKQYMELS |
|  | p11 | VTNNRHLGY |  | r11 | VMTYRPWLE |
|  | p12 | NRNRGTPGY |  | r12 | SGRVKMLLR |
|  | p13 | NTNNRRPGY |  | r13 | KRSGMWLNA |
|  | p14 | RKTTRRLGY |  | r14 | ASELPSFWR |
|  | p15 | VRTRHGLGY |  | r15 | SKYVRETPS |
| **Predicted Clones: Least-Similar** | p16 | NPNNTGRGY |  | r16 | IVHWMKSAQ |
|  | p17 | NKNTTGPGY |  | r17 | TQNYEFRWI |
|  | p18 | NKTTRHRGY |  | r18 | QMWNTPLEI |
|  | p19 | NTTNTGRGY |  | r19 | SIWRHTMNP |
|  | p20 | NNTNTGRGY |  | r20 | LEYQRTASD |
|  | p21 | RTTNTGLGY |  | r21 | DEHRYMIPN |
|  | p22 | RPTNTGLGY |  | r22 | WLPYEMNRL |
|  | p23 | VKTTTGLGY |  | r23 | VQLGSWNFA |
|  | p24 | VPTNNSYGY |  | r24 | PRAWSNLML |
|  | p25 | NTTNNSPGY |  | r25 | FVPWTEQMG |
