# Supplementary material for: Interrogating and Predicting Tolerated Sequence Diversity in Protein Folds: Application to E. elaterium Trypsin Inhibitor-II Cystine-Knot Miniprotein
Source: PLoS Comput Biol. 2009 Sep 4;5(9):e1000499. doi: 10.1371/journal.pcbi.1000499 (PMC2725296; doi:10.1371/journal.pcbi.1000499)
Supplement: Table S3 — Representative naturally-occurring knottins with loop sequences similar to those of enriched EL3-9 clones. C-terminal glycine-tyrosine doublets are shown in bold and the corresponding loop sequences are underlined. Clones whose underlined loop sequence is at least 50% homologous to the loop sequence of a clone from the enriched EL3-9 library are italicized and bolded. Sequences are listed according to their sub-family groupings in the KNOTTIN database (http://knottin.cbs.cnrs.fr/Knottins.php) and are named according to their UniprotKB/Swiss-Prot accession numbers. (0.07 MB DOC) [file pcbi.1000499.s003.doc]

**Table S3. Representative naturally-occurring knottins with loop sequences similar to those of enriched EL3-9 clones.** C-terminal glycine-tyrosine doublets are shown in bold and the corresponding loop sequences are underlined. Clones whose underlined loop sequence is at least 50% homologous to the loop sequence of a clone from the enriched EL3-9 library are italicized and bolded. Sequences are listed according to their sub-family groupings in the KNOTTIN database (<http://knottin.cbs.cnrs.fr/Knottins.php>) and are named according to their UniprotKB/Swiss-Prot accession numbers.

| **Sub-Family** | **Accession #** | **Sequence** |
| --- | --- | --- |
| Conotoxin 1 | ***Q9BP80*** | MKLTCVLIIAVLFLTACQLTTGEQKDHALRSTDKNSKLTRQCTPVG**GY**CSRHYHCCSNHCIKSIGRCVAH |
|  | ***Q9TVK3*** | VLIIAVLFLTACQLTTAETSSRGKQKHRALRSTDKNSRMSKRCTPPG**GY**CYHPDPCCSQVCNFPRKHCL |
|  | ***A9P3W1*** | VLIIAVLFLTACQLTTAVTSSRGEQKHRALRSTDKNSRLTKRCTPRN**GY**CYYRYFCCSRACNLTIKRCL |
|  | ***A9P3X5*** | VMIVAVLFLTACQLTTADTSSRGKQKYRVLRSTDKNSRLTRVCTPPE**GY**CTYHRDCCDLYCNKTTNVCLET |
|  | ***Q3YEF8*** | MEKLTSLLLVAALLMLTQTLIQGGGEDRPNKKFLQKIKSTAKRECTAPS**GY**CDYPEECCEVECGRHYCDWWY |
| Fungi 2 | B0DU64 | MHSFKFTRPFALVLAIVCLAIATPTPDVHADPGHGSGAVVHAVGNAPDSAVIKDTITTHLPAATSPTDKTLGGSTLEPDSSECFGLGSPCSFNSNCCS**GY**CLIIPPTIVLGFCYPK |
|  | A7EBW4 | MHLLQPRLIILLLFSLSTQIFASLTERKLCIKNGEVCHLTGESCCDGFKCALAHGGKANV**GY**CTESGFLLQDYYQVRLP |
| Insect | P83653 | CIKNGNGCQPNGSQGNCCS**GY**CHKQPGWVA**GY**CRRK |
| Antimicrobial | P83651 | CIKNGNGCQPDGSQGNCCSRYCHKEPGWVA**GY**CR |
| Plant | O81338 | MAKVSSSLLKFAIVLILVLSMSAIISAKCIKNGKGCREDQGPPFCCSGFCYRQVGWAR**GY**CKNR |
| Antimicrobial | P25403 | LPVAFLKFAIVLILFIAMSAMIEAQCIGNGGRCNENVGPPYCCSGFCLRQPGQGY**GY**CKNR |
| Plant Toxin | ***P62926*** | MASVKLASLIVLFATLGMFLTKNVGAASCNGVCSPFEMPPCGTSACRCIPVGLVV**GY**CRNPSGVFLRTNDEHPNLCESDADCRKKGSGNFGHYPNPDIEYGWCFASKSEAEDFFSKITPKDLLKSVSTA |
|  | Q6A1C8 | MAYVRLTHLVVFLLSTFSLFPMKKVGATDCSGICSPFEMPPCRSSDCRCIPIVLVG**GY**CINPISPAATKMVKEHPNLCHSHTDCTKKGSGSFCARYPNP |
| Scorpion 1 | ***P83400*** | MKFLYGTILIAFFLTVMIATHSEARCPPCFTTNPNMEADCRKCCGGR**GY**CASYQCICPGG |
| Spider | ***P85144*** | GGCINHGQPCDGDKNDCQCCRDN**GY**CNCDGIFGLKWNCKC |
|  | ***P81744*** | SCIDFGGDCDGEKDDCQCCRSN**GY**CSCYNLFGYLKSGCKCEVGTSAEFRRICRRKAKQCYNSDPDKCVSVYKPKRR |
|  | ***P33034*** | SCIDIGGDCDGEKDDCQCCRRN**GY**CSCYSLFGYLKSGCKCVVGTSAEFQGICRRKARQCYNSDPDKCESHNKPKRR |
|  | P61233 | CGTNRAWCRNAKDHCCCGYSCVKPIWASKPEDD**GY**CWKKFGGC |
| Serine | ***Q43667*** | MAAFVESARAGAGADEVIQLVSDGVNEYSEKMMEGVVACPRILMPCKVNDDCLRGCKCLSN**GY**CG |
| Protease | ***P10291*** | MVCPKILMKCKHDSDCLLDCVCLEDI**GY**CGVS |
| Inhibitor | ***P82408*** | SGSDGGVCPKILQRCRRDSDCPGACICRGN**GY**CG |
|  | ***P82410*** | QRACPRILKKCRRDSDCPGECICKEN**GY**CG |
|  | ***Q9S8D2*** | VGCPRILMKCKTDRDCLTGCTCKRN**GY**CG |
|  | ***P17680*** | GICPRILMECKRDSDCLAQCVCKRQ**GY**CG |
|  | ***P07853*** | HEERVCPRILMKCKKDSDCLAECVCLEH**GY**CG |
| Trematoda | Q5BQU9 | MKYINLMLTIILALSLETFHIIDACREIGEVCSKTVFDKCCGNTVCKLRGPFYGECVECLNSGERCWRNSECCS**GY**CRWFTCQD |
