# Supplementary material for: Interrogating and Predicting Tolerated Sequence Diversity in Protein Folds: Application to E. elaterium Trypsin Inhibitor-II Cystine-Knot Miniprotein
Source: PLoS Comput Biol. 2009 Sep 4;5(9):e1000499. doi: 10.1371/journal.pcbi.1000499 (PMC2725296; doi:10.1371/journal.pcbi.1000499)
Supplement: Dataset S1 — Recovered sequences of randomized loops from the unsorted and enriched EETI loop-substituted libraries. (0.37 MB DOC) [file pcbi.1000499.s004.doc]

**Dataset S1. Recovered sequences of randomized loops from the unsorted and enriched EETI loop-substituted libraries.**

| **Library** | **Unsorted Sequences** | **Enriched Sequences** |
| --- | --- | --- |
| **EL2-7** | GFRGFGQ | VPGGSGV |
|  | WCVGLPA | DPRVSGG |
|  | GRRWGFR | NVTLGMP |
|  | IVGAALY | RGTGSQS |
|  | VWGSRRG | GGPGSMG |
|  | VAGLWYW | RAGWDND |
|  | RRLLELV | GGPGKLT |
|  | LPDLSSL | MGSGKST |
|  | MLQKLWF | MPGGFGG |
|  | QGMAAGT | DPSRNRT |
|  | QCAASLG | DPGDRWG |
|  | RMRILLL | DPARKDA |
|  | FELCLWN | VPGVKGQ |
|  | GPYCGAW | SPNNGSG |
|  | VDVMACV | NVTWGTG |
|  | SKTWAAL | WPPGGGL |
|  | WAPPVTR | GGRAGQG |
|  | FYRPMFF | KGGSRSE |
|  | AMLDLLT | DHSAGRG |
|  | ARRVVSV | QWGVSGG |
|  | LDSYLQD | ELGSSGQ |
|  | ATLTVLV | DPLLGQG |
|  | LGFVIVG | DLRTGRG |
|  | GCSRSEH | NPAEGFF |
|  | SARVCGL | GFNGVGR |
|  | LGMRLWA | APHLGMG |
|  | AWLNSVR | GSVASGE |
|  | APVAVSL | GPPPSSE |
|  | KILLFHS | KPGFAGG |
|  | YMNAWGF | TPGLSGG |
|  | AAVSYLI | CRFCGSG |
|  | ALRCQEW | VPGRLDG |
|  | RFFTSGR | AGSGRGG |
|  | DQLAESV | GFRGFGQ |
|  | PCLGGPP | VGVGALP |
|  | VLDRSWW | VPGGRND |
|  | AYAQGKS | TPGQEAA |
|  | LGYLAEL | GSGPGSG |
|  | WAAWVSN | GGPGDMK |
|  | ELGVMSH | DPAKYGE |
|  | SKGMSWQ | QYGVPGG |
|  | FGGSVGQ | SGGDVEE |
|  | VKAQLWR | KVGLAGG |
|  | LRPETAG | VPGKRGR |
|  | LVLVPVL | DIGAGLG |
|  | TPGLSWG | GDKVGGG |
|  | PALTWQV | TPGAPGT |
|  | SFTVCVV | DSSVMGG |
|  | RTGHLLT | NLLNGSG |
|  | HVIMWPS | GGVWGNS |
|  | DWFLRDI | NMALGMG |
|  | TYMRIPR | DPAAQSQ |
|  |  | VPGRENE |
|  |  | GGGRARQ |
|  |  | GRADLPP |
|  |  | NPMLRGG |
|  |  | GGISGAR |
|  |  | GAVHGGG |
|  |  | EPGVEVT |
|  |  | GPGIGGT |
|  |  | APGLVNT |
| **EL2-9** | TLWRWLGDL | NSFAESHAA |
|  | VKCNRIVLV | VTLEGLRLP |
|  | SLYERGVLC | SSSLGSGCG |
|  | CLVRSLFLG | EPGLHSNSG |
|  | EACAGLCLC | DPASSLLYT |
|  | IPLLLPWEV | GKAGPTHLK |
|  | WLGFPVWTK | VLAFGESWG |
|  | MFLSRVINY | DYDRWDRGS |
|  | WLDLCLFLE | GATVRGGGG |
|  | GFVVRSSGM | GTLPGASRS |
|  | MCLLAAVWY | VPGMPIGFG |
|  | MLFLNRGKG | SKGLDSRSG |
|  | GVSCAMWDC | GPHSGGVGT |
|  | LYVKLLLLW | VPGGQGGSA |
|  | LGGVRLRCS | SSKDHSPGT |
|  | SELFLCFLV | AMSPLLNQG |
|  | YSLFGNALL | SPGIGSGKQ |
|  | LFLFGLLSR | SYGDGLGHS |
|  | FFLLVFHAS | GAVWGVPSL |
|  | LLYWSRWGF | GSGSSAWGG |
|  | QVFREILLL | AIMGILVRR |
|  | WIWSVLRFR | GSSASEESE |
|  | VLFMHRALF | GGTGDPVSG |
|  | ALLPVFLVV | RVRGGVLSP |
|  | TVSFYVAWP | DPMKGGALG |
|  | VASWHGPIR | SFGSKGAEG |
|  | SVLLASFEL | GRRGPGGNG |
|  | GAGRFVFVS | ERGAGPSGQ |
|  | LRPWMDEVA | GRWNGSVYS |
|  | SVWRAKVCV | SPELLGARP |
|  | GVLVRGSSW | GAMAGGRLP |
|  | LFALRLWWA | RSLRGVKTP |
|  | FFLVEKWTG | SLRGKFVRP |
|  | CLCSWGGKC | SSSGSGEMG |
|  | SVFCFLVLS | LSVGAVGSQ |
|  | PLCEWSLNY | REPGSGLAA |
|  | RINLAMVCG | GGAYGTLGV |
|  | GWWLVRLLS | GVVQGRRVA |
|  | VVWLLISVG | NPNAASSVV |
|  | VWSGWSSLF | RYSRGKGRG |
|  | ASGLLGSCK | GWVQGAFVS |
|  | WLWWRSGVL | TGDLGSSYG |
|  | GYAEVYWPL | LVSESGDSG |
|  | WHSCLYGLF | GVGDVSGNA |
|  | RLLGYLGGL | WSGNGESGG |
|  | LAGLLDVFY | EVGVGRLHG |
|  | VISGWLLEV | SHPGGVRSP |
|  | KFHLSRFMP | VAGGHHRGG |
|  | DLVMSRVVV | LPWKSTSSN |
|  | PNAIFVWSE | GLVGGLWMD |
|  |  | ISSRASVRQ |
|  |  | GFGNNASSG |
|  |  | APKLNLPYQ |
| **EL3-6** | HTKSKH | HFDSGT |
|  | QTANVR | HTRSHM |
|  | SRHSSL | HTKDGF |
|  | IPKLDT | NLRLHM |
|  | PQQYYE | NSYSRY |
|  | HTAPDH | DHNVGF |
|  | RHLITP | SNTSHT |
|  | PHSRRQ | DRLTRY |
|  | KPPPNY | DPTSGH |
|  | ANAIYS | DPRTMI |
|  | MPHSPS | FTQYHY |
|  | NSDDNN | DTVTNY |
|  | DPRPNQ | RLPDGM |
|  | VMLHES | RKRDNY |
|  | SPHPGR | DTRFNL |
|  | MPTAIC | CKPEGY |
|  | MSRSAQ | DQMTKY |
|  | PTHTHP | NLSHGV |
|  | PNQNER | HAQDGR |
|  | RTRNQH | HSESML |
|  | HIKVPE | NRSSLT |
|  | ERATTH | SDRSNL |
|  | TQPLYV | DSDVGI |
|  | PPTTPN | RLNTWT |
|  | KALANT | HLASSY |
|  | DSHKSP | NVRNKV |
|  | HTGVAH | LYSSGT |
|  | IFATGP | HKRTRF |
|  | GTYPRQ | QKLSGH |
|  | KQYENT | DIWSRH |
|  | PDPSNG | NQDDAT |
|  | TMFTHL | DQLNNN |
|  | GWKNAR | HYRTSL |
|  | YKDTTA | MRKNGY |
|  | SLKQTR | HTKFRM |
|  | TNLEGL | NPRSRF |
|  | TKRRGE | HTSAGA |
|  | MVKNQW | NQRYKI |
|  | TDEATG | NVLSKT |
|  | SMCPWT | NKASFT |
|  | DSKFAR | STPNGY |
|  | GVQSPR | QRVVGY |
|  | DTYTST | NKRAMK |
|  | HAEKTS | NRRFRY |
|  | STASSG | DRTSSR |
|  | SVVNTT | NAKSRT |
|  | PNRLPH | RNPAGT |
|  | GQNTTN | DQLSRI |
|  | SLPQYV | CNDYTC |
|  | PNEDIR | DPLVNF |
|  | HRDTSP | QTNTHT |
|  | RSTRSM | NANHGY |
|  | RPLRNV | DTIAQL |
|  |  | NTRSGT |
|  |  | NTDMNY |
|  |  | SSAVLT |
|  |  | DSAMNY |
|  |  | MSFSQY |
|  |  | HPQDRY |
|  |  | DGSPGT |
|  |  | NQDSNY |
|  |  | DPRTEY |
| **EL3-7** | STPENLL | RRGQRSL |
|  | DTKRPMQ | RTGVTRF |
|  | LTGYLST | MRSLPRY |
|  | NPRNHTI | LRGKHRF |
|  | PNNPTPN | ISTYCSP |
|  | AYDGPSV | ILSITGY |
|  | TPPVVLW | LSPNAGF |
|  | LLASRRR | GYPDRPF |
|  | QTYTHLC | DTLTGYC |
|  | YNAQNTG | STRVGIC |
|  | QKVHSKG | VGLAGGF |
|  | QPRLTFG | SATFCTQ |
|  | NQHFLAD | SSYPYGY |
|  | SPLASHS | DPASSTC |
|  | FRTHLDS | ISSGMGF |
|  | LDNIVAR | DLSSNWC |
|  | QTRSASH | LGLPLGV |
|  | TSTLPCD | VHSRHGY |
|  | PNPPMRT | NLRTRAC |
|  | QTPPMPL | TSTPHGY |
|  | HQAAYGN | RPNAHGY |
|  | HSPYSAL | SEKAPRY |
|  | FLVPLAD | FEAVCQT |
|  | RKRSCGL | QFPFCRT |
|  | QHALLPV | LRGTTRY |
|  | HCHTPRP | RKPPDGF |
|  | LYKAPHL | DTASSTC |
|  | PHTPWHF | SRSYCAQ |
|  | GPPLRPS | NHPPAGT |
|  | TLQSATW | LGGLLAY |
|  | EVYTLTW | RGTTPGY |
|  | GPPLVNQ | ISNYCAP |
|  | RTSIIPS | CRSRYKY |
|  | SNTRMRI | NPPANPF |
|  | FRHFNPA | ARESGFC |
|  | IRFQSTC | HPVHRLC |
|  | HMLRNAS | GLGPMRV |
|  | VNASHKP | LVGNPSY |
|  | DSSPQIF | RNASPHY |
|  | TPAVPGF | NENTGGF |
|  | QHRSSTS | DRNHGGY |
|  | IPYGHGL | TGRHQRY |
|  | RRELPPL | MDNYCRG |
|  | GPGALPY | DKSTDIC |
|  | VPFRSCY | CPTEGHC |
|  | LKPILGL | RQHLCAP |
|  | PTPASTN | RGGYRKY |
|  | HLAHNSY | IVGRKSF |
|  | VPEARTF | NKQGIGL |
|  | HGTSLPA | PNREPRY |
|  | LPFVRSH | RHSTPRY |
|  | MSGSLRE | NLNNTGY |
|  |  | KLRLIGY |
|  |  | RTYIYGT |
|  |  | HPGPTGY |
|  |  | NSLQGGF |
| **EL3-8** | SANSPKDD | LNSGTKRF |
|  | RRSLRTQL | IGNFNRAY |
|  | LHPTRANS | RLNGLRGE |
|  | SNPPHNNH | ILPTASGY |
|  | INSYGKSP | RRYNHVGM |
|  | IGRNDPAS | TQNTYYGY |
|  | IHTNLTNI | RGKTGISL |
|  | MRNFDPLQ | LKDGQTGH |
|  | SMPVETSM | VTRKFSAY |
|  | LEDPAGDR | IQDHGISF |
|  | PCGLSSNP | VPTENEKY |
|  | SYKVEGPS | RFRRYMGI |
|  | TSQSCIM | INNGQHGY |
|  | ISSPNTTH | VRNKDESY |
|  | TCVNLDMA | HSTPGGAF |
|  | HPFNKTTT | SGWKQRKF |
|  | MHSTRKTN | IKTQRLAY |
|  | TIASVTRS | AHHVMGDY |
|  | NLVLRPRH | CYSSIMGY |
|  | VDHYKCKG | VKPGRRGV |
|  | MHAQHLEP | IRNRRHGI |
|  | EMHSESCR | IPSRPPQY |
|  | IARRGSTR | MHRKRGRY |
|  | ARYRQQSY | RNNGKRKT |
|  | ASPRMNSM | MKHGNDKY |
|  | TALNARSM | NHGTHVSY |
|  | MDIPAQDC | RHDRGKGH |
|  | VEHLTSHG | NNRHPNAY |
|  | RHRNVGPR | VHSRGRRY |
|  | RGSMPTAT | RYTGSAGY |
|  | MGSQISSQ | RIARRHGW |
|  | PNLNTNKQ | RCTAHKCH |
|  | PHTHRFRN | NMIGRHSF |
|  | TSDGTGGQ | NGMQHNRF |
|  | IDIIQRKQ | SRHKHANY |
|  | PNKTYAHV | ISNRHNGW |
|  | SKVGAPVP | TTSAQGGT |
|  | MITCTHTI | ISSNKRST |
|  | SPPANPNI | VHSRDTAY |
|  | IGQTVARY | IKNRKRRY |
|  | RAPPPLSQ | RRTKGRSF |
|  | LRNNRASD | RRSQDAHY |
|  | RAIVRPQT | LPAAQGGT |
|  | ISYTTHAT | LSGRRFDW |
|  | IATHPQYH | RNDPQGRY |
|  | TNPRTSHL | MNRKNFGF |
|  | RTRNCHRT | VSPLRTGY |
|  | STLPGPIS | RLSSNPPT |
|  | RGTPKLTT | NHRLTSGY |
|  | ITKTIYAR | IHYEIGGR |
|  | PNRTQIKT | LRTSSHNY |
|  |  | GCSPNGCT |
|  |  | NHFTHISL |
|  |  | RARGEMKY |
|  |  | TYANTIGY |
|  |  | RGGGNVNY |
| **EL3-9** | KNHIDNATH | MRTRSNGGY |
|  | SYKNTPSGI | NPHTHGMGY |
|  | SNLKYMNRI | IYNKHGPRY |
|  | DSNTHYHYN | IGRKKTRMY |
|  | ARNVQSSFH | NKPRMNRGY |
|  | ASNSRKPNA | RDNARGKGT |
|  | NKPTRSIRK | LTHNRRVSY |
|  | VMNRRATVT | NKNFFRTGY |
|  | PKTNKKMSS | RITNETLSY |
|  | SDTRKAAHW | VRTRTGWDY |
|  | KTTTDISYT | RDMMNNMGY |
|  | GNNERYMNP | TFNEHGSGH |
|  | NSPHATNNH | VRSRGNFSY |
|  | AHTCNNESP | IRTSNQNWF |
|  | SHDPPNPLA | RVTPNSLSY |
|  | SNDPTKRYT | HQTDHNYGY |
|  | ESHMHKDPM | YRGTCGYKH |
|  | RYNTYDMKN | VPSNGTYGY |
|  | HGRKNCDIT | VNTNTGLGY |
|  | ANIQLTWTQ | RRAPYANGY |
|  | VEDKVTKHA | RKIHMKTGY |
|  | DISLIAKNN | VAYEKTLGY |
|  | RSTAEKRVN | SGGTNDTRY |
|  | LTTKHNTKN | NNRFGQTSY |
|  | RQISYAYTV | NTMHRTNGY |
|  | GNPDNTENN | NNRNRRAGY |
|  | NMASSTAYN | NLNPHGMSF |
|  | NPPIMVMTP | NRSRDKTAY |
|  | KRKPNRGPQ | NIRDSAPWY |
|  | IAHSSNINM | HMNHPRLGF |
|  | NKLSSHFTQ | NPTNRDISY |
|  | NNLNGRNST | NNKQSRRGY |
|  | NRNPMCKTL | NKITGRPGY |
|  | RWARTVSRS | VNARKSMGY |
|  | TSRVSTRNV | RPGNGTIRW |
|  | YYKPPSSTM | HKTGISPHY |
|  | KNSHMTIYR | RTNNGGPNY |
|  | CPQSKNIRT | NHVSRHRGY |
|  | LKKTQSTSK | IKVTKEDGY |
|  | TTITVNYKH | NMNGRTPGY |
|  | NSRHTLSIN | MKRNRYGAY |
|  | NPMNPITYV | VTNRSIYGY |
|  | EPNVEIRTD | KAHTRHISY |
|  | NNSYTDYDI | HTTTTDAGY |
|  | KRPRQNPPG | NRDIYNRGY |
|  | THTTTCEPY | VTNEGEQGY |
|  | SNSTHPDGN | NPTGEDVGY |
|  | MCTTHKKAK | NTNPDNKGY |
|  | ETLICTMSN | NRRANVDGF |
|  | EPPDPSERT | NDITDDTGY |
|  | HGQYTNTNL | KKYTNSISY |
|  | NTKTCPMFS | YRQRRVMGY |
|  | RGTRKHTNT |  |
|  | HTQNQSNSL |  |
|  | NRPKHRHDH |  |
|  | ENKTGGQPI |  |
