# Supplementary material for: Interrogating and Predicting Tolerated Sequence Diversity in Protein Folds: Application to E. elaterium Trypsin Inhibitor-II Cystine-Knot Miniprotein
Source: PLoS Comput Biol. 2009 Sep 4;5(9):e1000499. doi: 10.1371/journal.pcbi.1000499 (PMC2725296; doi:10.1371/journal.pcbi.1000499)
Supplement: Dataset S2 — Raw covariance scores for the unsorted EL3-9 library calculated using the OMES, SCA, ELSC, MI, and McBASC scoring functions. (0.09 MB DOC) [file pcbi.1000499.s005.doc]

**Dataset S2. Raw covariance scores for the unsorted EL3-9 library calculated using the OMES, SCA, ELSC, MI, and McBASC scoring functions.**

| **(i)** | **(j)** | **OMES** | **SCA** | **ELSC** | **MI** | **McBASC** |
| --- | --- | --- | --- | --- | --- | --- |
| 1 | 2 | 0.8463238 | 17.068625 | 1.5040773 | 1.3664876 | 0.0813021 |
| 1 | 3 | 0.8887572 | 36.544404 | 5.4240685 | 1.1743749 | 0.0592769 |
| 1 | 4 | 0.7294779 | 23.483346 | 1.7917594 | 1.2634725 | 0.0398189 |
| 1 | 5 | 0.8281022 | 22.999183 | 4.1588830 | 1.4317552 | 0.0343712 |
| 1 | 6 | 0.7609329 | 16.311348 | 5.3752784 | 1.1854727 | 0.0962322 |
| 1 | 7 | 0.7169278 | 18.598104 | 2.9549102 | 1.2971133 | 0.0232058 |
| 1 | 8 | 0.8513347 | 24.263808 | 3.1780538 | 1.2572662 | 0.0689565 |
| 1 | 9 | 0.7241936 | 16.993967 | 0.5596157 | 1.2807854 | 0.0357295 |
| 2 | 3 | 0.9104409 | 22.253332 | 4.2766661 | 1.2105666 | 0.0303177 |
| 2 | 4 | 0.7785850 | 11.307981 | 3.8066624 | 1.2253984 | 0.0277969 |
| 2 | 5 | 0.8421328 | 16.008270 | 4.4465651 | 1.4090926 | 0.0013792 |
| 2 | 6 | 0.9407798 | 24.845515 | 4.6821312 | 1.3300291 | 0.0069220 |
| 2 | 7 | 0.8451166 | 19.848824 | 4.5643481 | 1.3487165 | 0.0483086 |
| 2 | 8 | 0.8921510 | 16.292529 | 0.1823215 | 1.3088695 | 0.0079719 |
| 2 | 9 | 0.9155202 | 29.479589 | 3.1416861 | 1.4005865 | 0.0156451 |
| 3 | 4 | 0.8534074 | 32.731833 | 3.8066624 | 1.2252600 | 0.0161216 |
| 3 | 5 | 0.8586916 | 42.770601 | 4.3412046 | 1.2945216 | 0.0870733 |
| 3 | 6 | 0.9217383 | 36.849639 | 2.7850112 | 1.2121822 | 0.0216954 |
| 3 | 7 | 0.8120444 | 21.718907 | 1.4508328 | 1.2649686 | 0.0500041 |
| 3 | 8 | 0.8005648 | 17.871444 | 3.4657359 | 1.1167568 | 0.0342896 |
| 3 | 9 | 0.8186042 | 25.056827 | 2.8415815 | 1.2238854 | 0.0212964 |
| 4 | 5 | 0.9666317 | 39.801244 | 3.3603753 | 1.5073955 | 0.0204958 |
| 4 | 6 | 0.7610240 | 18.763603 | 4.9008204 | 1.2176703 | 0.0653475 |
| 4 | 7 | 0.8637026 | 18.651340 | 2.2617630 | 1.4035767 | 0.0021372 |
| 4 | 8 | 0.9308263 | 21.138256 | 5.3062855 | 1.2647086 | 0.0558689 |
| 4 | 9 | 0.7648733 | 31.004833 | 4.5122321 | 1.2821599 | 0.0316236 |
| 5 | 6 | 0.8755466 | 19.627212 | 3.8712010 | 1.4200519 | 0.0194776 |
| 5 | 7 | 0.9814139 | 51.384256 | 2.2617630 | 1.6059583 | 0.0885521 |
| 5 | 8 | 0.8058718 | 16.151529 | 2.8903717 | 1.3246265 | 0.0607436 |
| 5 | 9 | 0.8226812 | 33.302115 | 2.3025850 | 1.4410989 | 0.0167989 |
| 6 | 7 | 0.8052113 | 31.162114 | 4.2234216 | 1.3503321 | 0.0404999 |
| 6 | 8 | 0.8288083 | 20.523523 | 2.4849066 | 1.2609745 | 0.0912600 |
| 6 | 9 | 0.7328717 | 31.698570 | 2.1972245 | 1.2443269 | 0.0222820 |
| 7 | 8 | 0.7452623 | 33.341448 | 2.3025850 | 1.2642504 | 0.0323691 |
| 7 | 9 | 0.7838921 | 59.596087 | 3.5710964 | 1.3961343 | 0.0060411 |
| 8 | 9 | 0.7700209 | 15.706985 | 0.2876820 | 1.2231673 | 0.0366192 |
