# Supplementary material for: Interrogating and Predicting Tolerated Sequence Diversity in Protein Folds: Application to E. elaterium Trypsin Inhibitor-II Cystine-Knot Miniprotein
Source: PLoS Comput Biol. 2009 Sep 4;5(9):e1000499. doi: 10.1371/journal.pcbi.1000499 (PMC2725296; doi:10.1371/journal.pcbi.1000499)
Supplement: Dataset S3 — Raw covariance scores for the enriched EL3-9 library calculated using the OMES, SCA, ELSC, MI, and McBASC scoring functions. Scores shown in bold are those whose corresponding z-scores are greater than 2. (0.10 MB DOC) [file pcbi.1000499.s006.doc]

**Dataset S3. Raw covariance scores for the enriched EL3-9 library calculated using the OMES, SCA, ELSC, MI, and McBASC scoring functions.** Scores shown in bold are those whose corresponding z-scores are greater than 2.

| **(i)** | **(j)** | **OMES** | **SCA** | **ELSC** | **MI** | **McBASC** |
| --- | --- | --- | --- | --- | --- | --- |
| 1 | 2 | 0.5830109 | 14.279366 | 2.4567357 | 0.8515960 | 0.0321273 |
| 1 | 3 | 0.6785389 | 19.392408 | 4.2766661 | 0.8241828 | 0.0287617 |
| 1 | 4 | 0.9333750 | 13.010220 | 4.0253516 | 0.9317325 | 0.0324543 |
| 1 | 5 | 0.8723828 | 17.193407 | 4.8283137 | 0.9508340 | 0.0187796 |
| 1 | 6 | 0.6075330 | 17.151729 | 2.9267394 | 0.8033883 | 0.0086415 |
| 1 | 7 | 0.8909877 | 21.023427 | 5.1929568 | 1.0912239 | **0.0979014** |
| 1 | 8 | 0.5707783 | 46.221523 | -0.0430173 | 0.6021111 | 0.0716267 |
| 1 | 9 | 0.2139280 | 25.813445 | 0.1941560 | 0.2666439 | 0.0372113 |
| 2 | 3 | 0.9439292 | 25.680820 | 5.1059454 | 1.1689660 | 0.0075264 |
| 2 | 4 | **0.9833295** | **76.091678** | **6.1862086** | 1.2713123 | 0.0211098 |
| 2 | 5 | 0.7117091 | 29.850376 | 4.2766661 | 1.1207387 | 0.0864372 |
| 2 | 6 | 0.8692250 | 33.161118 | 5.1239639 | 1.1647686 | 0.0084934 |
| 2 | 7 | 0.7724169 | 24.672479 | 4.7874917 | 1.2659876 | 0.0196393 |
| 2 | 8 | 0.4347974 | 29.531015 | 3.2580965 | 0.6271959 | 0.0460763 |
| 2 | 9 | 0.3026001 | **69.616480** | 1.8191584 | 0.3502465 | **0.1076451** |
| 3 | 4 | 0.8600364 | 20.780216 | 4.4308167 | 1.1625893 | 0.0050199 |
| 3 | 5 | 0.9033909 | 40.591764 | 4.2766661 | 1.1969567 | 0.0660112 |
| 3 | 6 | 0.8954540 | **51.562717** | 3.7170503 | 1.1623393 | 0.0168878 |
| 3 | 7 | 0.8519572 | 26.744042 | 4.9698133 | 1.2622272 | 0.0260188 |
| 3 | 8 | 0.6265361 | 43.331125 | 0.9808292 | 0.5870578 | 0.0403679 |
| 3 | 9 | 0.2767694 | **94.292458** | 2.5123056 | 0.2174898 | 0.0281847 |
| 4 | 5 | 0.8031975 | **61.288257** | 3.4293682 | 1.1777435 | 0.0880604 |
| 4 | 6 | 0.8644742 | 38.380929 | 2.1927702 | 1.1785169 | 0.0261838 |
| 4 | 7 | 0.8522416 | 21.518351 | 3.6243409 | 1.3395895 | 0.0237572 |
| 4 | 8 | 0.5150773 | **68.080612** | 3.5582011 | 0.5928285 | 0.0529885 |
| 4 | 9 | 0.2651342 | 27.309548 | 1.3862943 | 0.3251704 | 0.0101787 |
| 5 | 6 | **0.9817933** | 33.179157 | 4.0898902 | 1.2259534 | 0.0064135 |
| 5 | 7 | 0.7741237 | 16.385457 | 2.9957322 | 1.2839159 | 0.0238286 |
| 5 | 8 | 0.4295061 | 18.558862 | 0.2513144 | 0.6310681 | 0.0146271 |
| 5 | 9 | 0.2745789 | 28.468435 | 1.3862943 | 0.3333995 | 0.0033257 |
| 6 | 7 | 0.8075784 | 35.286661 | 4.2766661 | 1.2912238 | 0.0001064 |
| 6 | 8 | 0.4985775 | 41.129953 | 4.5390303 | 0.5929228 | 0.0127077 |
| 6 | 9 | 0.3411185 | **127.92934** | 5.0949545 | 0.2565489 | 0.0517872 |
| 7 | 8 | 0.6671028 | **73.996600** | 5.7186710 | 0.7461297 | 0.0578086 |
| 7 | 9 | 0.2272985 | **92.759852** | -0.4855078 | 0.3431542 | 0.01062177 |
| 8 | 9 | 0.1167500 | 12.845318 | 0.4054651 | 0.1829580 | 0.02012486 |
