# Supplementary material for: Interrogating and Predicting Tolerated Sequence Diversity in Protein Folds: Application to E. elaterium Trypsin Inhibitor-II Cystine-Knot Miniprotein
Source: PLoS Comput Biol. 2009 Sep 4;5(9):e1000499. doi: 10.1371/journal.pcbi.1000499 (PMC2725296; doi:10.1371/journal.pcbi.1000499)
Supplement: Dataset S4 — Predicted nine-amino acid sequences for substitution into EETI loop 3. (0.07 MB DOC) [file pcbi.1000499.s007.doc]

**Dataset S4. Predicted nine-amino acid sequences for substitution into EETI loop 3.**

1 NRNRRRRGY

2 NRNRRHRGY

3 NRNRNSRGY

4 NRNRTGRGY

5 NRNRHGRGY

6 NRNRKTRGY

7 NRNRGTRGY

8 NKNTRRRGY

9 NKNTRHRGY

10 NKNTNSRGY

11 NKNTTGRGY

12 NKNTHGRGY

13 NKNTKTRGY

14 NKNTGTRGY

15 NTNNRRRGY

16 NTNNRHRGY

17 NTNNNSRGY

18 NTNNTGRGY

19 NTNNHGRGY

20 NTNNKTRGY

21 NTNNGTRGY

22 NNNNRRRGY

23 NNNNRHRGY

24 NNNNNSRGY

25 NNNNTGRGY

26 NNNNHGRGY

27 NNNNKTRGY

28 NNNNGTRGY

29 NPNNRRRGY

30 NPNNRHRGY

31 NPNNNSRGY

32 NPNNTGRGY

33 NPNNHGRGY

34 NPNNKTRGY

35 NPNNGTRGY

36 NRNRRRTGY

37 NRNRRHTGY

38 NRNRNSTGY

39 NRNRTGTGY

40 NRNRHGTGY

41 NRNRKTTGY

42 NRNRGTTGY

43 NKNTRRTGY

44 NKNTRHTGY

45 NKNTNSTGY

46 NKNTTGTGY

47 NKNTHGTGY

48 NKNTKTTGY

49 NKNTGTTGY

50 NTNNRRTGY

51 NTNNRHTGY

52 NTNNNSTGY

53 NTNNTGTGY

54 NTNNHGTGY

55 NTNNKTTGY

56 NTNNGTTGY

57 NNNNRRTGY

58 NNNNRHTGY

59 NNNNNSTGY

60 NNNNTGTGY

61 NNNNHGTGY

62 NNNNKTTGY

63 NNNNGTTGY

64 NPNNRRTGY

65 NPNNRHTGY

66 NPNNNSTGY

67 NPNNTGTGY

68 NPNNHGTGY

69 NPNNKTTGY

70 NPNNGTTGY

71 RRNRRRLGY

72 RRNRRHLGY

73 RRNRNSLGY

74 RRNRTGLGY

75 RRNRHGLGY

76 RRNRKTLGY

77 RRNRGTLGY

78 RKNTRRLGY

79 RKNTRHLGY

80 RKNTNSLGY

81 RKNTTGLGY

82 RKNTHGLGY

83 RKNTKTLGY

84 RKNTGTLGY

85 RTNNRRLGY

86 RTNNRHLGY

87 RTNNNSLGY

88 RTNNTGLGY

89 RTNNHGLGY

90 RTNNKTLGY

91 RTNNGTLGY

92 RNNNRRLGY

93 RNNNRHLGY

94 RNNNNSLGY

95 RNNNTGLGY

96 RNNNHGLGY

97 RNNNKTLGY

98 RNNNGTLGY

99 RPNNRRLGY

100 RPNNRHLGY

101 RPNNNSLGY

102 RPNNTGLGY

103 RPNNHGLGY

104 RPNNKTLGY

105 RPNNGTLGY

106 VRNRRRLGY

107 VRNRRHLGY

108 VRNRNSLGY

109 VRNRTGLGY

110 VRNRHGLGY

111 VRNRKTLGY

112 VRNRGTLGY

113 VKNTRRLGY

114 VKNTRHLGY

115 VKNTNSLGY

116 VKNTTGLGY

117 VKNTHGLGY

118 VKNTKTLGY

119 VKNTGTLGY

120 VTNNRRLGY

121 VTNNRHLGY

122 VTNNNSLGY

123 VTNNTGLGY

124 VTNNHGLGY

125 VTNNKTLGY

126 VTNNGTLGY

127 VNNNRRLGY

128 VNNNRHLGY

129 VNNNNSLGY

130 VNNNTGLGY

131 VNNNHGLGY

132 VNNNKTLGY

133 VNNNGTLGY

134 VPNNRRLGY

135 VPNNRHLGY

136 VPNNNSLGY

137 VPNNTGLGY

138 VPNNHGLGY

139 VPNNKTLGY

140 VPNNGTLGY

141 VRNRRRYGY

142 VRNRRHYGY

143 VRNRNSYGY

144 VRNRTGYGY

145 VRNRHGYGY

146 VRNRKTYGY

147 VRNRGTYGY

148 VKNTRRYGY

149 VKNTRHYGY

150 VKNTNSYGY

151 VKNTTGYGY

152 VKNTHGYGY

153 VKNTKTYGY

154 VKNTGTYGY

155 VTNNRRYGY

156 VTNNRHYGY

157 VTNNNSYGY

158 VTNNTGYGY

159 VTNNHGYGY

160 VTNNKTYGY

161 VTNNGTYGY

162 VNNNRRYGY

163 VNNNRHYGY

164 VNNNNSYGY

165 VNNNTGYGY

166 VNNNHGYGY

167 VNNNKTYGY

168 VNNNGTYGY

169 VPNNRRYGY

170 VPNNRHYGY

171 VPNNNSYGY

172 VPNNTGYGY

173 VPNNHGYGY

174 VPNNKTYGY

175 VPNNGTYGY

176 NRNRRRPGY

177 NRNRRHPGY

178 NRNRNSPGY

179 NRNRTGPGY

180 NRNRHGPGY

181 NRNRKTPGY

182 NRNRGTPGY

183 NKNTRRPGY

184 NKNTRHPGY

185 NKNTNSPGY

186 NKNTTGPGY

187 NKNTHGPGY

188 NKNTKTPGY

189 NKNTGTPGY

190 NTNNRRPGY

191 NTNNRHPGY

192 NTNNNSPGY

193 NTNNTGPGY

194 NTNNHGPGY

195 NTNNKTPGY

196 NTNNGTPGY

197 NNNNRRPGY

198 NNNNRHPGY

199 NNNNNSPGY

200 NNNNTGPGY

201 NNNNHGPGY

202 NNNNKTPGY

203 NNNNGTPGY

204 NPNNRRPGY

205 NPNNRHPGY

206 NPNNNSPGY

207 NPNNTGPGY

208 NPNNHGPGY

209 NPNNKTPGY

210 NPNNGTPGY

211 NRTRRRRGY

212 NRTRRHRGY

213 NRTRNSRGY

214 NRTRTGRGY

215 NRTRHGRGY

216 NRTRKTRGY

217 NRTRGTRGY

218 NKTTRRRGY

219 NKTTRHRGY

220 NKTTNSRGY

221 NKTTTGRGY

222 NKTTHGRGY

223 NKTTKTRGY

224 NKTTGTRGY

225 NTTNRRRGY

226 NTTNRHRGY

227 NTTNNSRGY

228 NTTNTGRGY

229 NTTNHGRGY

230 NTTNKTRGY

231 NTTNGTRGY

232 NNTNRRRGY

233 NNTNRHRGY

234 NNTNNSRGY

235 NNTNTGRGY

236 NNTNHGRGY

237 NNTNKTRGY

238 NNTNGTRGY

239 NPTNRRRGY

240 NPTNRHRGY

241 NPTNNSRGY

242 NPTNTGRGY

243 NPTNHGRGY

244 NPTNKTRGY

245 NPTNGTRGY

246 NRTRRRTGY

247 NRTRRHTGY

248 NRTRNSTGY

249 NRTRTGTGY

250 NRTRHGTGY

251 NRTRKTTGY

252 NRTRGTTGY

253 NKTTRRTGY

254 NKTTRHTGY

255 NKTTNSTGY

256 NKTTTGTGY

257 NKTTHGTGY

258 NKTTKTTGY

259 NKTTGTTGY

260 NTTNRRTGY

261 NTTNRHTGY

262 NTTNNSTGY

263 NTTNTGTGY

264 NTTNHGTGY

265 NTTNKTTGY

266 NTTNGTTGY

267 NNTNRRTGY

268 NNTNRHTGY

269 NNTNNSTGY

270 NNTNTGTGY

271 NNTNHGTGY

272 NNTNKTTGY

273 NNTNGTTGY

274 NPTNRRTGY

275 NPTNRHTGY

276 NPTNNSTGY

277 NPTNTGTGY

278 NPTNHGTGY

279 NPTNKTTGY

280 NPTNGTTGY

281 RRTRRRLGY

282 RRTRRHLGY

283 RRTRNSLGY

284 RRTRTGLGY

285 RRTRHGLGY

286 RRTRKTLGY

287 RRTRGTLGY

288 RKTTRRLGY

289 RKTTRHLGY

290 RKTTNSLGY

291 RKTTTGLGY

292 RKTTHGLGY

293 RKTTKTLGY

294 RKTTGTLGY

295 RTTNRRLGY

296 RTTNRHLGY

297 RTTNNSLGY

298 RTTNTGLGY

299 RTTNHGLGY

300 RTTNKTLGY

301 RTTNGTLGY

302 RNTNRRLGY

303 RNTNRHLGY

304 RNTNNSLGY

305 RNTNTGLGY

306 RNTNHGLGY

307 RNTNKTLGY

308 RNTNGTLGY

309 RPTNRRLGY

310 RPTNRHLGY

311 RPTNNSLGY

312 RPTNTGLGY

313 RPTNHGLGY

314 RPTNKTLGY

315 RPTNGTLGY

316 VRTRRRLGY

317 VRTRRHLGY

318 VRTRNSLGY

319 VRTRTGLGY

320 VRTRHGLGY

321 VRTRKTLGY

322 VRTRGTLGY

323 VKTTRRLGY

324 VKTTRHLGY

325 VKTTNSLGY

326 VKTTTGLGY

327 VKTTHGLGY

328 VKTTKTLGY

329 VKTTGTLGY

330 VTTNRRLGY

331 VTTNRHLGY

332 VTTNNSLGY

333 VTTNTGLGY

334 VTTNHGLGY

335 VTTNKTLGY

336 VTTNGTLGY

337 VNTNRRLGY

338 VNTNRHLGY

339 VNTNNSLGY

340 VNTNTGLGY

341 VNTNHGLGY

342 VNTNKTLGY

343 VNTNGTLGY

344 VPTNRRLGY

345 VPTNRHLGY

346 VPTNNSLGY

347 VPTNTGLGY

348 VPTNHGLGY

349 VPTNKTLGY

350 VPTNGTLGY

351 VRTRRRYGY

352 VRTRRHYGY

353 VRTRNSYGY

354 VRTRTGYGY

355 VRTRHGYGY

356 VRTRKTYGY

357 VRTRGTYGY

358 VKTTRRYGY

359 VKTTRHYGY

360 VKTTNSYGY

361 VKTTTGYGY

362 VKTTHGYGY

363 VKTTKTYGY

364 VKTTGTYGY

365 VTTNRRYGY

366 VTTNRHYGY

367 VTTNNSYGY

368 VTTNTGYGY

369 VTTNHGYGY

370 VTTNKTYGY

371 VTTNGTYGY

372 VNTNRRYGY

373 VNTNRHYGY

374 VNTNNSYGY

375 VNTNTGYGY

376 VNTNHGYGY

377 VNTNKTYGY

378 VNTNGTYGY

379 VPTNRRYGY

380 VPTNRHYGY

381 VPTNNSYGY

382 VPTNTGYGY

383 VPTNHGYGY

384 VPTNKTYGY

385 VPTNGTYGY

386 NRTRRRPGY

387 NRTRRHPGY

388 NRTRNSPGY

389 NRTRTGPGY

390 NRTRHGPGY

391 NRTRKTPGY

392 NRTRGTPGY

393 NKTTRRPGY

394 NKTTRHPGY

395 NKTTNSPGY

396 NKTTTGPGY

397 NKTTHGPGY

398 NKTTKTPGY

399 NKTTGTPGY

400 NTTNRRPGY

401 NTTNRHPGY

402 NTTNNSPGY

403 NTTNTGPGY

404 NTTNHGPGY

405 NTTNKTPGY

406 NTTNGTPGY

407 NNTNRRPGY

408 NNTNRHPGY

409 NNTNNSPGY

410 NNTNTGPGY

411 NNTNHGPGY

412 NNTNKTPGY

413 NNTNGTPGY

414 NPTNRRPGY

415 NPTNRHPGY

416 NPTNNSPGY

417 NPTNTGPGY

418 NPTNHGPGY

419 NPTNKTPGY

420 NPTNGTPGY
