# Supplementary material for: Interrogating and Predicting Tolerated Sequence Diversity in Protein Folds: Application to E. elaterium Trypsin Inhibitor-II Cystine-Knot Miniprotein
Source: PLoS Comput Biol. 2009 Sep 4;5(9):e1000499. doi: 10.1371/journal.pcbi.1000499 (PMC2725296; doi:10.1371/journal.pcbi.1000499)
Supplement: Dataset S5 — Multiple sequence alignment of predicted EETI loop 3 sequences containing four or more common motifs. Numbers correspond to clone numbers as assigned in Dataset S4. The alignment was generated with ClustalW v.2.0.10. (0.05 MB DOC) [file pcbi.1000499.s008.doc]

**Dataset S5. Multiple sequence alignment of predicted EETI loop 3 sequences containing four or more common motifs.** Numbers correspond to clone numbers as assigned in Dataset S4. The alignment was generated with ClustalW v.2.0.10.

1 NRNRRRRGY 9

2 NRNRRHRGY 9

5 NRNRRRTGY 9

6 NRNRRHTGY 9

17 NRNRRRPGY 9

18 NRNRRHPGY 9

21 NRTRRRRGY 9

22 NRTRRHRGY 9

25 NRTRRRTGY 9

26 NRTRRHTGY 9

37 NRTRRRPGY 9

38 NRTRRHPGY 9

39 NRTRKTPGY 9

40 NRTRGTPGY 9

23 NRTRKTRGY 9

24 NRTRGTRGY 9

27 NRTRKTTGY 9

28 NRTRGTTGY 9

3 NRNRKTRGY 9

4 NRNRGTRGY 9

7 NRNRKTTGY 9

8 NRNRGTTGY 9

19 NRNRKTPGY 9

20 NRNRGTPGY 9

9 VRNRRRLGY 9

10 VRNRRHLGY 9

13 VRNRRRYGY 9

14 VRNRRHYGY 9

29 VRTRRRLGY 9

30 VRTRRHLGY 9

33 VRTRRRYGY 9

34 VRTRRHYGY 9

11 VRNRKTLGY 9

12 VRNRGTLGY 9

15 VRNRKTYGY 9

16 VRNRGTYGY 9

31 VRTRKTLGY 9

32 VRTRGTLGY 9

35 VRTRKTYGY 9

36 VRTRGTYGY 9

*.* **
