# Supplementary material for: Interrogating and Predicting Tolerated Sequence Diversity in Protein Folds: Application to E. elaterium Trypsin Inhibitor-II Cystine-Knot Miniprotein
Source: PLoS Comput Biol. 2009 Sep 4;5(9):e1000499. doi: 10.1371/journal.pcbi.1000499 (PMC2725296; doi:10.1371/journal.pcbi.1000499)
Supplement: Dataset S6 — Multiple sequence alignment of predicted EETI loop 3 sequences containing three common motifs. Numbers correspond to clone numbers as assigned in Dataset S4. The alignment was generated with ClustalW v.2.0.10. (0.06 MB DOC) [file pcbi.1000499.s009.doc]

**Dataset S6. Multiple sequence alignment of predicted EETI loop 3 sequences containing three common motifs.** Numbers correspond to clone numbers as assigned in Dataset S4. The alignment was generated with ClustalW v.2.0.10.

60 NKNTRRPGY 9

127 NKTTRRPGY 9

61 NKNTRHPGY 9

128 NKTTRHPGY 9

4 NKNTRRRGY 9

71 NKTTRRRGY 9

5 NKNTRHRGY 9

72 NKTTRHRGY 9

15 NKNTRRTGY 9

82 NKTTRRTGY 9

16 NKNTRHTGY 9

83 NKTTRHTGY 9

27 RKNTRRLGY 9

28 RKNTRHLGY 9

94 RKTTRRLGY 9

95 RKTTRHLGY 9

38 VKNTRRLGY 9

39 VKNTRHLGY 9

105 VKTTRRLGY 9

106 VKTTRHLGY 9

49 VKNTRRYGY 9

50 VKNTRHYGY 9

116 VKTTRRYGY 9

117 VKTTRHYGY 9

6 NKNTKTRGY 9

7 NKNTGTRGY 9

73 NKTTKTRGY 9

74 NKTTGTRGY 9

29 RKNTKTLGY 9

30 RKNTGTLGY 9

96 RKTTKTLGY 9

97 RKTTGTLGY 9

40 VKNTKTLGY 9

107 VKTTKTLGY 9

41 VKNTGTLGY 9

108 VKTTGTLGY 9

51 VKNTKTYGY 9

118 VKTTKTYGY 9

52 VKNTGTYGY 9

119 VKTTGTYGY 9

62 NKNTKTPGY 9

129 NKTTKTPGY 9

63 NKNTGTPGY 9

130 NKTTGTPGY 9

17 NKNTKTTGY 9

18 NKNTGTTGY 9

84 NKTTKTTGY 9

85 NKTTGTTGY 9

8 NTNNRRRGY 9

9 NTNNRHRGY 9

64 NTNNRRPGY 9

65 NTNNRHPGY 9

75 NTTNRRRGY 9

76 NTTNRHRGY 9

131 NTTNRRPGY 9

132 NTTNRHPGY 9

19 NTNNRRTGY 9

86 NTTNRRTGY 9

20 NTNNRHTGY 9

87 NTTNRHTGY 9

10 NTNNKTRGY 9

66 NTNNKTPGY 9

21 NTNNKTTGY 9

77 NTTNKTRGY 9

133 NTTNKTPGY 9

88 NTTNKTTGY 9

11 NTNNGTRGY 9

67 NTNNGTPGY 9

22 NTNNGTTGY 9

78 NTTNGTRGY 9

134 NTTNGTPGY 9

89 NTTNGTTGY 9

31 RTNNRRLGY 9

32 RTNNRHLGY 9

98 RTTNRRLGY 9

99 RTTNRHLGY 9

42 VTNNRRLGY 9

43 VTNNRHLGY 9

109 VTTNRRLGY 9

110 VTTNRHLGY 9

53 VTNNRRYGY 9

54 VTNNRHYGY 9

120 VTTNRRYGY 9

121 VTTNRHYGY 9

33 RTNNKTLGY 9

34 RTNNGTLGY 9

100 RTTNKTLGY 9

101 RTTNGTLGY 9

44 VTNNKTLGY 9

111 VTTNKTLGY 9

45 VTNNGTLGY 9

112 VTTNGTLGY 9

55 VTNNKTYGY 9

122 VTTNKTYGY 9

56 VTNNGTYGY 9

123 VTTNGTYGY 9

1 NRNRNSRGY 9

12 NRNRNSTGY 9

57 NRNRNSPGY 9

124 NRTRNSPGY 9

68 NRTRNSRGY 9

79 NRTRNSTGY 9

35 VRNRNSLGY 9

102 VRTRNSLGY 9

46 VRNRNSYGY 9

113 VRTRNSYGY 9

2 NRNRTGRGY 9

3 NRNRHGRGY 9

58 NRNRTGPGY 9

59 NRNRHGPGY 9

13 NRNRTGTGY 9

14 NRNRHGTGY 9

69 NRTRTGRGY 9

125 NRTRTGPGY 9

80 NRTRTGTGY 9

70 NRTRHGRGY 9

126 NRTRHGPGY 9

81 NRTRHGTGY 9

36 VRNRTGLGY 9

37 VRNRHGLGY 9

103 VRTRTGLGY 9

104 VRTRHGLGY 9

47 VRNRTGYGY 9

114 VRTRTGYGY 9

48 VRNRHGYGY 9

115 VRTRHGYGY 9

23 RRNRRRLGY 9

24 RRNRRHLGY 9

90 RRTRRRLGY 9

91 RRTRRHLGY 9

25 RRNRKTLGY 9

92 RRTRKTLGY 9

26 RRNRGTLGY 9

93 RRTRGTLGY 9

. **
